# Rapid Global Expansion of Invertebrate Fisheries: Trends, Drivers, and Ecosystem Effects

**DOI:** 10.1371/journal.pone.0014735

**Published:** 2011-03-08

**Authors:** Sean C. Anderson, Joanna Mills Flemming, Reg Watson, Heike K. Lotze

**Affiliations:** 1 Department of Biology, Dalhousie University, Halifax, Nova Scotia, Canada; 2 Department of Mathematics and Statistics, Dalhousie University, Halifax, Nova Scotia, Canada; 3 Fisheries Centre, University of British Columbia, Vancouver, British Columbia, Canada; National Oceanic and Atmospheric Administration/National Marine Fisheries Service/Southwest Fisheries Science Center, United States of America

## Abstract

**Background:**

Worldwide, finfish fisheries are receiving increasing assessment and regulation, slowly leading to more sustainable exploitation and rebuilding. In their wake, invertebrate fisheries are rapidly expanding with little scientific scrutiny despite increasing socio-economic importance.

**Methods and Findings:**

We provide the first global evaluation of the trends, drivers, and population and ecosystem consequences of invertebrate fisheries based on a global catch database in combination with taxa-specific reviews. We also develop new methodologies to quantify temporal and spatial trends in resource status and fishery development. Since 1950, global invertebrate catches have increased 6-fold with 1.5 times more countries fishing and double the taxa reported. By 2004, 34% of invertebrate fisheries were over-exploited, collapsed, or closed. New fisheries have developed increasingly rapidly, with a decrease of 6 years (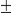
3 years) in time to peak from the 1950s to 1990s. Moreover, some fisheries have expanded further and further away from their driving market, encompassing a global fishery by the 1990s. 71% of taxa (53% of catches) are harvested with habitat-destructive gear, and many provide important ecosystem functions including habitat, filtration, and grazing.

**Conclusions:**

Our findings suggest that invertebrate species, which form an important component of the basis of marine food webs, are increasingly exploited with limited stock and ecosystem-impact assessments, and enhanced management attention is needed to avoid negative consequences for ocean ecosystems and human well-being.

## Introduction

Global finfish catches from capture fisheries peaked in the 1980s and have declined or remained stable since the early 1990s, yet global invertebrate catches have continued to climb [1]. Although some invertebrate fisheries have existed for centuries [2]–[4], many others have commenced or rapidly expanded over the past 2–3 decades [5], [6]. Today, shrimp has the largest share of the total value of internationally-traded fishery products (17% in 2006, including aquaculture), followed by salmon (11%), groundfish (10%), tuna (8%), and cephalopods (4%) [1]. In several ways, invertebrate fisheries represent a new frontier in marine fisheries: they provide an alternative source of animal protein for people, job opportunities in harvesting and processing, and substantial economic opportunities for communities due to their high value and expanding markets [1], [5], [6]. Yet, while finfish fisheries [7] and some more established invertebrate fisheries [8]–[11] have received increasing assessment, regulation, and rebuilding, many invertebrate fisheries do not get the same level of attention or care. They are typically not assessed, not monitored, and often unregulated [1], [2], [5], [6], [12], which threatens their sustainable development despite their increasing social, economic, and high ecological importance [6], [13].

The increase in invertebrate fisheries is in part a response to declining finfish catches that caused many fishermen to switch to new target species, often further down the food web [6], [14] although in many regions lower-trophic-level fisheries were added without declines in higher-trophic-level fisheries [15]. At the same time, the abundance and availability of many invertebrates may have increased due to release from formerly abundant finfish predators [16]. Once thought to be particularly resistant to over-exploitation [17], an increasing number of historical [3], [4] and recent invertebrate fisheries [2], [5], [12] tell a different story. Thus, in light of their increasing importance, we evaluated the current status, as well as the spatial and temporal trends of invertebrate fisheries around the world. Further, we aimed to assess their underlying drivers, and population and ecosystem consequences.

Unfortunately, stock assessments and research survey data that are available to evaluate many finfish populations [7] are often lacking for invertebrates [6], [12], [13]. Therefore, we used the Sea Around Us Project's catch database (Text S1) as the best available data source to analyze temporal and spatial trends in invertebrate fisheries on a global scale. It consists largely of a quality-checked version of the Food and Agriculture Organization's (FAO) catch database supplemented by regional and reconstructed datasets covering 302 invertebrate species or species groups (taxa) over 175 countries from 1950–2004 [18]. Wherever possible we have corroborated the observed patterns with recent taxa-specific global reviews (Text S1).

## Results and Discussion

**Figure 1:**
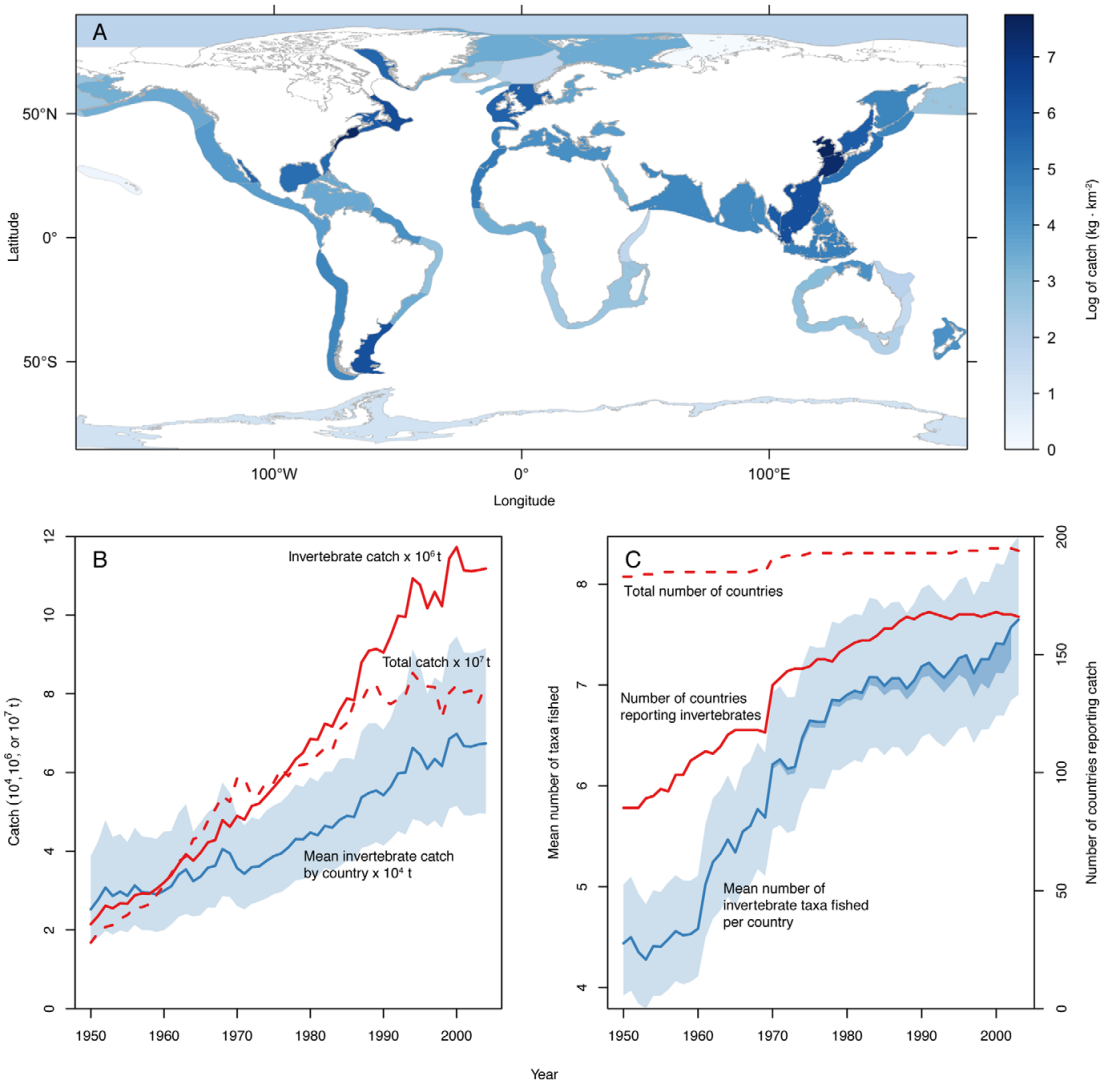
Spatial and temporal trends in catch, species diversity and countries involved in global invertebrate fisheries. (A) Mean annual invertebrate catch in each Large Marine Ecosystem (LME) from 2000–2004. (B) Trends in invertebrate catch globally (red) and per country (mean and standard error assuming a log-normal distribution, blue). Trends in all finfish and invertebrate catch (total catch, dashed red) are included as a reference. (C) Trends in the number of countries reporting catch of invertebrates (solid red) and of all finfish and invertebrate species (total, dashed red, as a reference) since the 1950s, and number of invertebrate taxa (taxonomic groups or species) fished by country (mean and standard error assuming a negative binomial distribution, blue). Thickness of dark blue line approximates false increase due to increased reporting precision (Text S1).

**Figure 2:**
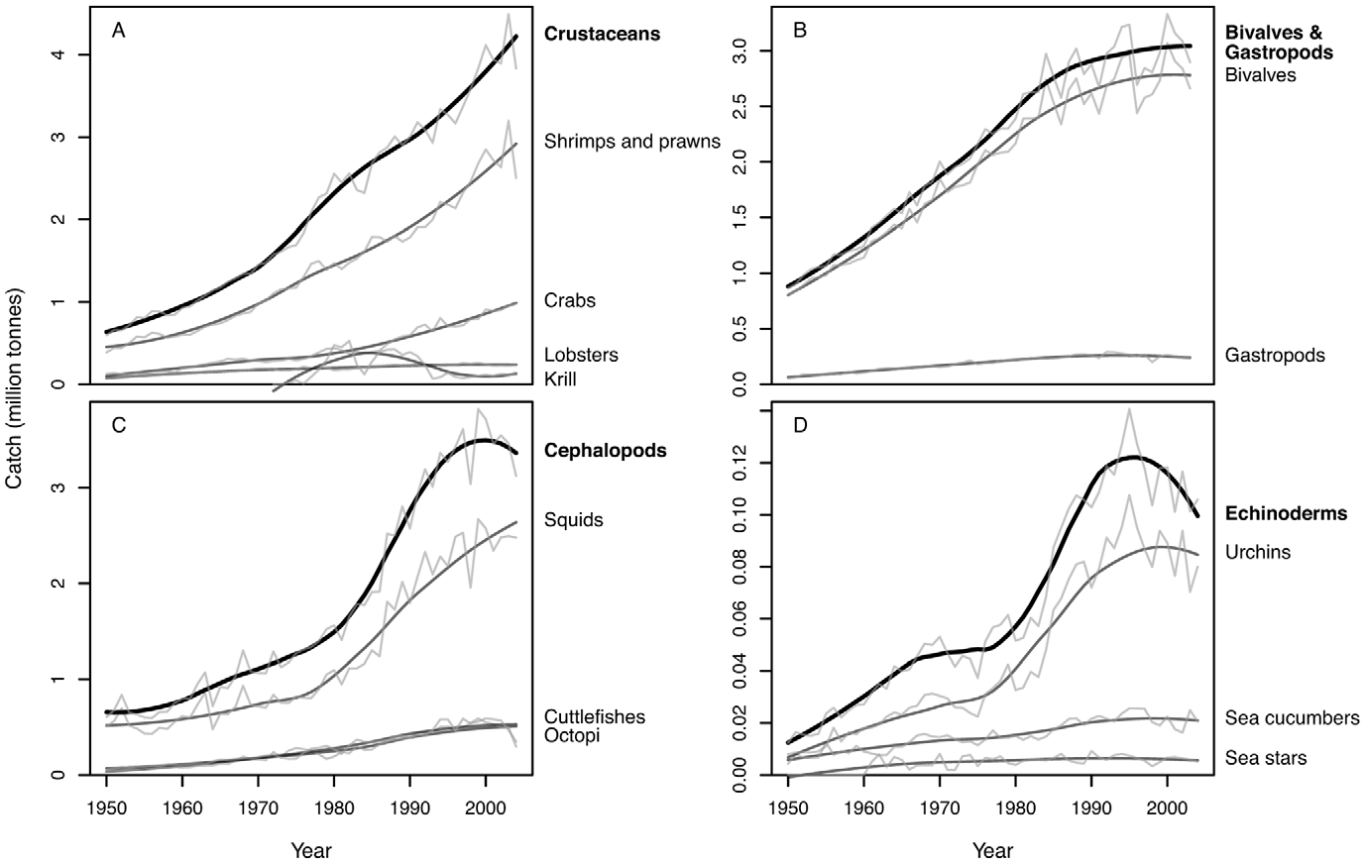
Expansion of invertebrate catch since the 1950s across taxa. (A) crustaceans, (B) bivalves and gastropods, (C) cephalopods, and (D) echinoderms. Upper lines indicate total catch for each group and underlying lines indicate catch for subgroups. Dark lines represent smooth estimates obtained from a loess smoother (smoothing span 50% of the data). Light lines indicate the unfiltered catch trends.

Since 1950, invertebrate fisheries have rapidly expanded on multiple scales, and today operate around the world (Fig. 1A). In 2000–2004, the highest concentrations of catch per unit area by Large Marine Ecosystem (LME) were in the Yellow Sea, East China Sea, and the Northeast U.S. Continental Shelf, followed by the Newfoundland-Labrador Shelf, South China Sea, and Patagonian Shelf. The bulk of the catch in these areas consisted of bivalves, shrimps, squids, and crabs (Table S1). Catches for all 4 of the larger invertebrate taxonomic groups (crustaceans, bivalves, cephalopods, and echinoderms) were heavily concentrated in the Yellow Sea and East China Sea (Fig. S1). In addition, catches for crustaceans were highly concentrated off the Newfoundland-Labrador Shelf, bivalves on the Northeast U.S. Continental Shelf, cephalopods off the Patagonian Shelf, and echinoderms off the Humboldt Current (Fig. S1). Since 1950, the total reported catch of invertebrates has steadily increased 6-fold from 2 to 12 million t (Fig. 1B). In comparison, the catch of invertebrates and finfish combined increased 5-fold over the same period, beginning to decline in the late 1980s [14], [19]. The increase in invertebrate catch is not driven by only a few countries, as the average catch per country has more than doubled (Fig. 1B). Also, in 2004 there were 1.5 times more countries fishing for twice as many invertebrate taxa compared to 1950 (Fig. 1C). This is in contrast to all finfish and invertebrate fisheries combined, where the number of countries reporting catch has been largely stable over the past 50 years (Fig. 1C). Although increasing trends in invertebrate fisheries may be partly explained by increasing precision in reporting (Fig. S2), there are clear underlying trends of expansion by catch, country, and taxa (Figs. 1B, 1C, 2). This is corroborated by studies on individual fisheries where assessments or effort data are available [20], [21].

The increase in invertebrate fisheries is driven not by a few major target species, but instead by increasing catch trends across all taxonomic groups (Fig. 2, Fig. S3). While catches have increased continuously since the 1950s for more traditionally fished crustaceans and bivalves, they rapidly increased in the 1980s and 1990s for often newly targeted cephalopods and echinoderms. Thus, already existing fisheries expanded and new fisheries were developed for species that had not been commercially fished before. Although overall catch trends for invertebrate fisheries paint a picture of continuing expansion (Fig. 1B), catches in several groups (e.g., octopus and echinoderms) have slowed or declined in recent years (Fig. 2B and 2D). The picture of universal increase changes even more drastically if we look at individual invertebrate fisheries by country. Here, some countries are still expanding their catches while others peaked long ago (Fig. S4, see also [5], [21]).

**Figure 3:**
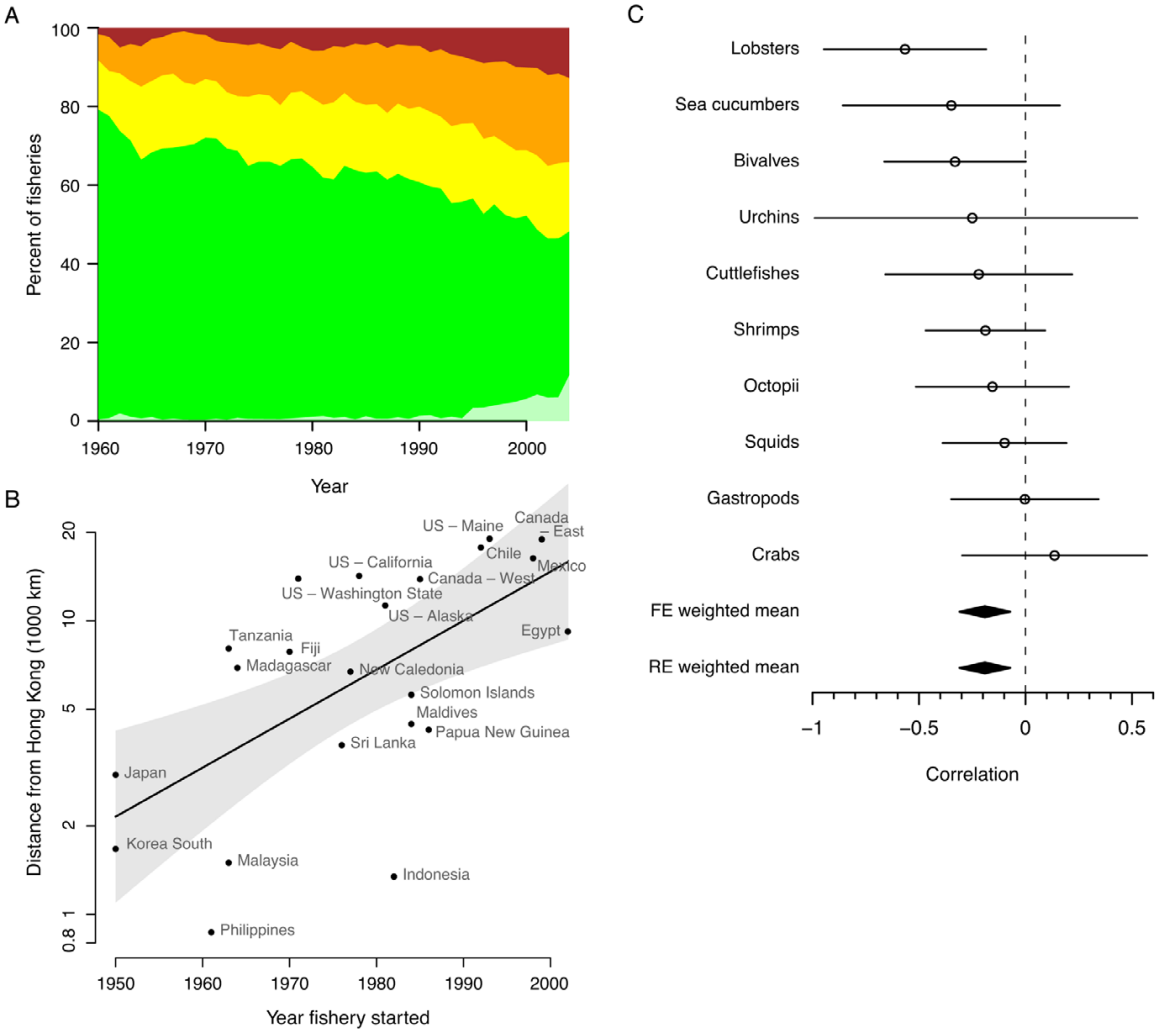
Status, drivers, and rate of development of invertebrate fisheries. (A) Estimated status of invertebrate fisheries over time as expanding (green), fully exploited (yellow), over-exploited or restrictively managed (orange), and collapsed or closed (dark red/brown) based on catch data. Light green indicates fisheries with less than 10 years of data at year of assessment. These fisheries were not evaluated. (B) Distance from Hong Kong vs. year of first peak in catch for sea cucumber fisheries in different countries. Line represents least squares regression (r = 0.62, p = 0.002), and shaded area represents 95% confidence interval. Note that the analysis presented here differs from that reported in [21], see Text S1. (C) Meta-analysis of correlation between fishery initiation year and time to peak catch across 10 invertebrate taxonomic groups. Dots represent median correlation coefficients, lines represent 95% confidence intervals, and diamonds represent fixed (FE) and random effect (RE) pooled estimates (Text S1).

Based on individual catch trajectories, we assessed the current status and patterns of depletion of invertebrate fisheries. To do this, we modified a technique of Froese and Kesner-Reyes [22] to estimate the exploitation status of each invertebrate fishery from catch data (Fig. S5). Our modifications overcome previous weaknesses of this method by accounting for high variability in catch, spurious peak catch years, and fisheries that are still expanding (see *Materials and Methods* and Text S1). Our results suggest that half of the fisheries had peaked as of 2004 (Fig. 3A), with 18% fully exploited, 21% over-exploited or restrictively managed, and 13% collapsed or closed with little difference across functional groups (Fig. S6). This, combined with evidence of an increasing number of countries reporting catch and an increasing number of taxonomic groups targeted (Fig. 1C), indicates that the globally increasing invertebrate catches (Fig. 1B) are likely supplied by new taxa or new countries entering the fishery. In some invertebrate fisheries, such as many sea cucumber fisheries [21], decreasing catch trends have been directly related to population declines; however, we do not suggest that catch trends are generally good indicators of population status or have been driven solely by high exploitation pressure. Declines in catch can also have natural (e.g., recruitment failure due to climate) and other human-related (e.g., changing markets, restrictive management) drivers that can act in conjunction with each other [23].

Strong global markets may drive the expansion and serial depletion of some fisheries over space and time [3], [5], [24], particularly given the increasing availability of efficient fishing gear, rapid global transport, and the incentive to preferentially fish profitable marine resources [25]. If a fishery is declining in one region, fishing companies move into other regions, usually further away, to supply the demand of global buyers [5]. Some new invertebrate fisheries have a single strong market, as shown for sea urchins [5], where the global catch is related to the value of the Japanese Yen [26]. For other taxa, single driving markets are less obvious. For example, squid has 3 main importing nations (Japan, Italy, and Spain), while others have even more (Text S1). However, the vast majority of global sea cucumber catch is exported to Hong Kong (64% by volume between 1950–2004) or nearby Asian countries and the value of sea cucumbers has risen dramatically in recent decades [27]. To test whether spatial expansion has occurred, we used least-squares regression to compare the great-circle distance from Hong Kong with the year of peak sea cucumber catch for each country (Text S1) and found a significantly positive relationship (r = 0.62, p = 0.002, Fig. 3B). Given the generally poor stock status of sea cucumber fisheries [27], this may indicate a strong driving market where fisheries are sequentially exploited in relation to transportation distance. Such serial exploitation can have strong negative social and ecosystem consequences [5].

If markets and prices increase, new fisheries may develop more rapidly over time. To test this, we compared the time when invertebrate fisheries began or expanded with the time when they reached an initial peak in catch (see *Materials and Methods* and Text S1). We used an initial rather than overall peak in catch trajectories to treat new and old fisheries equally. Despite uncertainty within the results for individual taxa, we found a significant overall reduction in time to peak for newer fisheries (Fig. 3C). This corresponds to an approximate decrease of 6 years (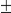
3 years, 95% confidence interval) in time to peak when comparing the 1950s to the 1990s. We suggest this may be a result of better fishing technology combined with growing demand due to the increasing global human population, changes in diet preferences (e.g., the rise of sushi restaurants in Western countries), declines in finfish fisheries, as well as more and more smaller fisheries being exploited, facilitated by global transport. We note that a pattern of serial depletion and substitution of other species within each investigated taxonomic group could mask peaks in catch [21] causing us to underestimate the rapid development of fisheries. Where a peak in catch represents a peak in fishery productivity, it is unlikely that management and research can keep up with this rate of expansion to ensure sustainable development [5], [6].

**Figure 4:**
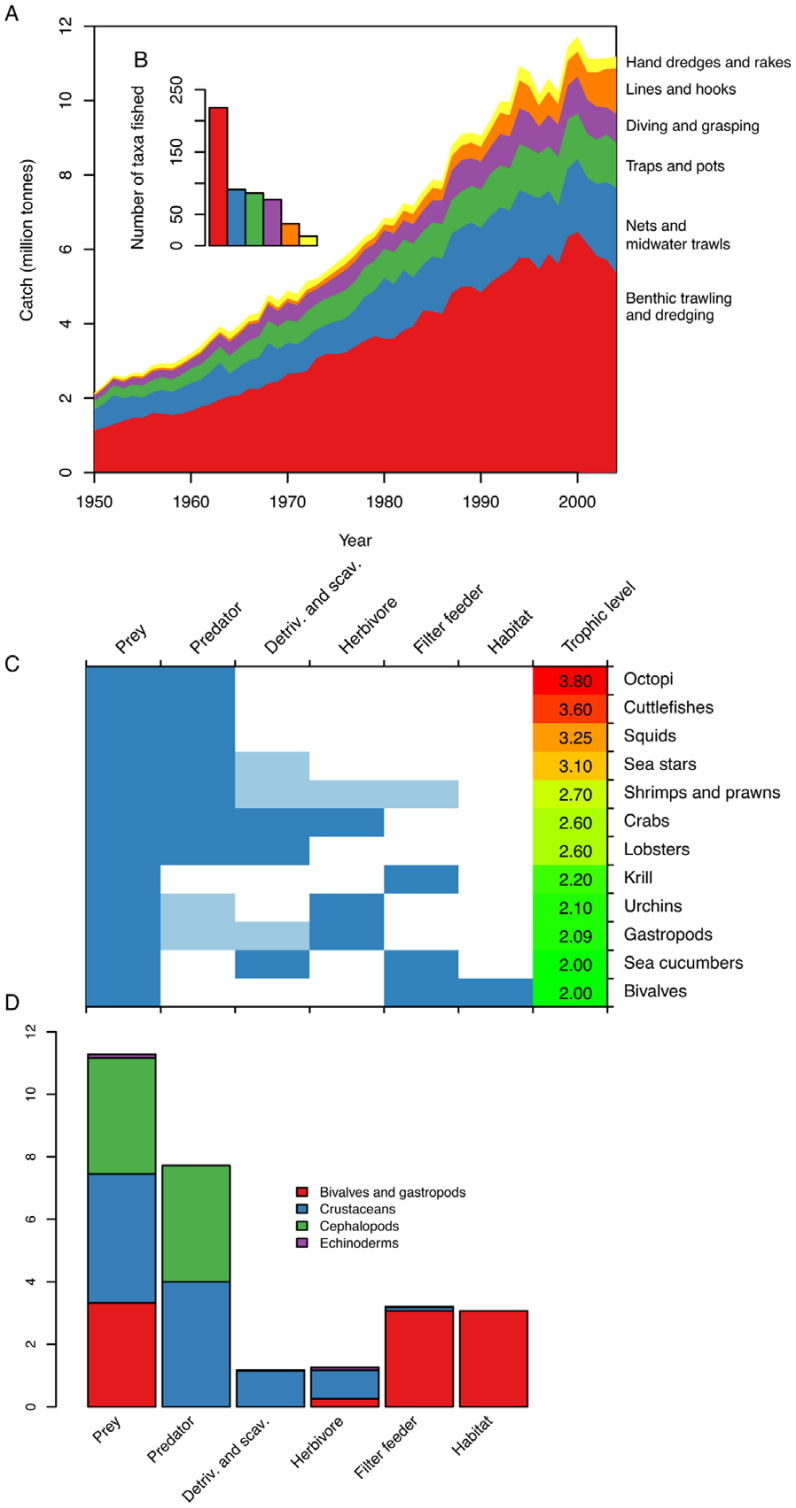
Potential ecosystem effects of invertebrate fisheries. Habitat impacts expressed as (A) total invertebrate catch and (B, inset in A) number of taxa fished by different gear types. (C) Ecosystem role of invertebrate taxa belonging to different functional groups and trophic levels (Text S1). Dark and light blue indicate primary and secondary roles respectively (Text S1). (D) Removal impact expressed as total catch removed by functional group as categorized in C (only the primary roles were included).

The rapid expansion, and in some cases serial depletion, of global invertebrate fisheries may have strong ecosystem consequences due to the method of fishing and the functional roles invertebrates play in marine ecosystems. In 2000–2004, 53% of invertebrate catch by volume and 71% by taxa fished were caught by benthic trawling and dredging gear with these proportions remaining relatively stable since the 1950s (Fig. 4A, B). This is largely driven by benthic trawling for crustacean and cephalopod species and dredging for bivalves. In comparison, benthic trawling and dredging accounted for only 20% of global finfish catch (57% of taxa, 2000–2004 mean). Such gear has substantive negative impacts on most benthic habitats and communities by destroying three-dimensional structure, impacting spawning and nursery grounds, altering benthic community composition, and reducing future biomass, production, and species richness [28]–[30]. Moreover, together with mid-water trawls, benthic trawls and dredges can incur a substantial portion of incidental by-catch [31].

Beyond the predator-prey roles that most finfish play in marine ecosystems, invertebrates have more diverse functions and more often provide essential ecosystem services such as maintaining water quality [32], [33], regenerating nutrients [34], providing nursery and foraging habitat [35], and preventing algal overgrowth through grazing [36] (Fig. 4C). It has been shown that the massive historic removal of oysters, such as in Chesapeake Bay, was associated with increases in eutrophication and hypoxia [33]. We aggregated mean catch per year from 2000–2004 by functional groups to assess the potential removal impact (Fig. 4D, Table S4) (see *Materials and Methods* and Text S1). All invertebrate taxa form potentially important roles as prey for higher trophic levels while most cephalopods and crustaceans also perform predatory roles. Especially bivalve, but also krill and some sea cucumber fisheries, represent a substantial removal by volume (3 million t/year) of filter feeders. We estimate the removal of bivalves alone to equate to a loss of 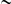
11 million Olympic-sized swimming pools (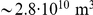) in filtering capacity per day in 2000–2004 (Text S1). In addition, many bivalves form beds, banks, or reefs that structure the seafloor and provide important habitat [35]. Invertebrate fisheries further remove 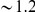 million t of detritivores and scavengers and 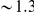 million t of herbivores annually. Although recruitment and re-growth will compensate for some of these losses, the direct and indirect short- and long-term ecosystem effects of these removals are largely unknown.

Our results demonstrate that despite overall increasing catches, diversity, and country participation in global invertebrate fisheries, there is strong evidence that the underlying trends in many individual fisheries are less optimistic. Our new and more robust analysis of catch trends suggests that an increasing percentage of invertebrate fisheries may be over-exploited, collapsed, or closed. Some invertebrate fisheries, such as the rock lobster fishery in western Australia, have existed for a long time and are well-managed [9], yet even there factors beyond the management system, such as climate change, can present major challenges. However, the same is not true for many newer fisheries like those for sea urchins [5], [12] and sea cucumbers [27] for which new fisheries develop further away from their market(s) and at an increasingly rapid rate, likely driven by strong market forces. This means that global industries, markets, and free trade may enable the rapid expansion of new fisheries before scientists and managers can step in and make sensible decisions to secure the long-term, sustainable use of these resources [5]. On the one hand, we risk losing some of the last remaining viable and financially lucrative fisheries, bringing financial and social hardship to a large number of small communities dependent on these fisheries for income or food. At the same time, the population and ecosystem consequences of many invertebrate fisheries are largely unknown and unassessed [6], although there are notable exceptions [8]–[11]. Whereas there is increasing assessment, regulation, and rebuilding of finfish fisheries to achieve more sustainable harvesting [7], many invertebrate fisheries do not enjoy the same awareness or attention. Many of the described patterns are reminiscent of an earlier phase in finfish fisheries during which the rate of finding new fishing areas, new target species, and more efficient gears masked overall catch trends [14]. However, because of improved industrial fishing gear and global networks that allow rapid and accessible transport, we may be progressing through invertebrate fishery phases even faster.

In order to prevent further uncontrolled expansion and instead aim for a more sustainable development of invertebrate fisheries, we highlight the need for a global perspective in their management combined with local assessment, monitoring, and enforcement of fisheries regulations. A global perspective is essential to identify roving buyers, monitor foreign investments, and consider CITES (U.N. Convention on International Trade in Endangered Species) listing where appropriate [5]. Also, the displacement of fishing effort from highly- to less-regulated regions and illegal, unreported, and underreported (IUU) catches requires global regulations in invertebrates and finfish fisheries alike [7]. On a regional and local scale, stock assessments are infrequently or not performed for many invertebrate fisheries and often lack adequate knowledge on the species biology, population status, and response to exploitation [6]. Invertebrates are rarely monitored in research trawl surveys [7], and independent research surveys to assess population trends, by-catch, and habitat impacts of invertebrate fisheries are rarely done for many newer fisheries [5], [6], [12]. Based on such limited knowledge, the sustainable exploitation of invertebrates for fisheries may be difficult to achieve [13].

In contrast, after many decades of increasing exploitation and fish stock depletion, concerted management efforts in several regions around the world achieved the reverse: a reduction in overall exploitation rate and an increase in stock biomass in several finfish fisheries [7]. This was achieved by a combination of management tools adapted to local conditions as well as strong legislation and enforcement. Similar measures can be implemented in invertebrate fisheries to prevent current and future trajectories of depletion [10]. As an example, the addition of co-management and property rights in Chilean artisanal gastropod fisheries solved many overexploitation concerns, substantially increasing catch per unit effort and mean individual size [11]. Similarly, the New Zealand rock lobster fishery was on a path of declining abundance before a reduction in effort and season length substantially increased abundance, catch rates, and profitability [8]. Such successes provide a great opportunity to inform the management of other newer fisheries. It is our hope that increasing awareness of the ecological and economic importance of invertebrates may spur more rigorous scientific assessment, precautionary management, and sustainable exploitation to ensure long-term resilience of invertebrate populations, ocean ecosystems, and human well-being.

## Materials and Methods

### Temporal and spatial catch trends

The Sea Around Us catch data are recorded by (i) country and (ii) LME for which catches are assigned to 30×30 minute cells [37]. We mapped spatial patterns in global catches as the mean annual invertebrate catch per 100 km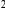 in each LME from 2000–2004 (Text S1). We also mapped spatial patterns of global catches for 4 major taxonomic groups (Fig. S1). Temporal trends from 1950–2004 were derived for overall invertebrate catch, total finfish and invertebrate catch, and mean invertebrate catch per country per year. Wherever possible, we corroborated the observed trends with recent taxa-specific global reviews (Text S1).

Globally, over 1200 taxonomic groups and species are reported caught in invertebrate or finfish fisheries, however, only the top species are recorded individually by the Sea Around Us Project with the remaining aggregated into groups such as “crustaceans” and “molluscs”. We obtained catch data for a total of 302 “taxa” (including 213 species) and analyzed the number of taxa fished over time, the number of countries fishing, and catch trends for 4 aggregated taxonomic groups (crustaceans, bivalves, echinoderms, and cephalopods), and 12 species groups. To some extent, the increasing diversity of taxa is a function of the increasing taxonomic precision of reporting over time. Therefore, we approximated the degree to which the increasing diversity reflected a true trend of an increasing number of species being targeted by fisheries (Fig. S2, Text S1).

The designation of countries can change over time; however, such changes are reflected in the overall number of countries reporting any catch for both finfish and invertebrate species, which we included as a reference (Fig. 1C). Overall, the country designation variation was small compared to the much larger changes of increasing participation in invertebrate fisheries. Nonetheless, we took this overall reporting trend into account and scaled the number of countries reporting catch of different invertebrate taxonomic and species groups to the total number of countries fishing finfish or invertebrates in any given year (Fig. S3).

### Assessment of fishery status from catch trends

Previous attempts have been made to categorize the status of fisheries using catch data [1], [22]. However, these approaches (i) can incorrectly categorize a fishery as over-exploited or collapsed due to single or multiple years of anomalous high catch and (ii) require all non-declining fisheries to be categorized as fully-exploited by the end of the time series. We developed a modified method for defining fishery status designed to take into account these two shortcomings by (i) applying a loess smoother to downweight outlying values and (ii) allowing fisheries to remain expanding at the end of the time series (Text S1). Further we assessed fishery status dynamically year-by-year to treat old and new fisheries equally (Fig. S5, Text S1). Dynamically evaluating the loess smoothed catches each year, a fishery was considered “expanding” until there were at least 5 years since a maximum in smoothed catch. A fishery was then defined as “fully-exploited”. If smoothed catch increased again, a fishery would be classified as “expanding”. When smoothed catch was less than 50% of a previous peak in catch, a fishery was defined as “over-exploited”. A fishery was defined as “collapsed or closed” when smoothed catch fell below 10% of peak catch. We demonstrate the robustness of our approach with simulated data (Fig. S7, Fig. S8, Text S1).

### Correlation of distance from Hong Kong

We were interested in testing whether some invertebrate fisheries followed a pattern of spatial expansion and depletion over time as has been shown for sea urchins [5]. Few species, however, have a single strong market, making such detection difficult. For sea cucumbers, the majority of catch is imported by Hong Kong [21], [38]. Thus we used the great circle distance between Hong Kong and the largest cities in each country with a sea cucumber fishery as a proxy for the transportation distance between the importing and exporting nations. We separated the US and Canadian east and west coasts because they are of substantially differing distances from Hong Kong. We then related log-transformed distance to the starting year of each fishery, which we calculated as the year at which loess smoothed catch passed 10% of its first peak in catch (Fig. S9A, Text S1). See the subsequent section *Analysis of fishery development time* for a description of the calculation of the first peak in catch. We cross-checked the starting years with published records (Table S2).

### Analysis of fishery development time

We tested whether there was evidence that newer fisheries were developing more rapidly over time by checking for a relationship between when invertebrate fisheries began and the time when they achieved their first peak in catch. Here, a fishery was defined as 1 of the 12 larger taxonomic groupings as reported by country (Fig. 2). We excluded sea stars and krill due to the limited number of countries with substantial fisheries. To focus on substantial fisheries, we discarded all fisheries less than 1000 t/year, except for lower-volume sea urchin and sea cucumber fisheries for which we used a minimum catch of 250 t/year. Our overall conclusions were invariant to choices of cutoffs from 500–2000 t (Text S1).

Catch trajectories can have multiple smaller local peaks together with an overall peak. If we naively calculated the peak catch from the entire available catch trajectory we would be more likely to be measuring local peaks (rather than overall peaks) with fisheries that started more recently. To avoid this time based bias we developed a dynamic assessment method that calculated the time it took for each fishery to develop to the first peak in catch (Fig. S9, Text S1). For each year, we fit a loess curve to the data up to that year and a fishery was considered to have reached a peak in catch if the following conditions were true: (1) a maximum in catch occurred and was less than 3 years from the end of the catch series at that step, (2) a maximum in catch occurred that was at least 500 t for most taxa or 125 t for echinoderms, and (3) the maximum in catch was at least 10% greater than the catch at the current time step. For fisheries that had yet to reach a peak in catch by the end of the time series, we assigned simulated values based on the observed times to a peak in catch for other fisheries (Fig. S10). We repeatedly re-assigned these simulated values and evaluated the correlation between year of initiation and time to the first peak in catch. See Text S1 for further details.

### Ecosystem effects

To assess the potential habitat effects of different invertebrate fisheries, we calculated the total invertebrate catch and the number of taxa fished by different gear types. The Sea Around Us Project reports 19 types of fishing gear for invertebrates, which we grouped into 6 broader groups based on their potential habitat impact (Table S3, Text S1). To evaluate the potential food web and ecosystem impacts of different invertebrate fisheries, we assigned primary and secondary functional groups to larger species groupings according to the primary literature and reference books (Table S4). Trophic levels were obtained from the Sea Around Us Project, where they were mainly derived from Froese and Pauly [39]. We then assessed the overall removal of each primary functional group as the total catch per functional group averaged over 2000–2004. This does not include renewal of resources via recruitment and re-growth. We also estimated the consequence of removing filter-feeding bivalves from the ocean in more detail, in terms of their capacity to filter water, using filtration rates reported in the literature. We converted these values into Olympic-sized swimming pools for comparison (Text S1).

## Supporting Information

Text S1 Supplementary description of the methods.
(0.15 MB PDF)

Figure S1 Mean annual invertebrate catch by taxonomic group in each Large Marine Ecosystem (LME) from 2000–2004.
(1.13 MB TIF)

Figure S2 Effects of taxonomic precision in reporting on predicted trends in diversity of invertebrates fished. (A) Increasing reporting of invertebrate taxa fished divided into species level (blue), larger grouping level (green), and combined (red). Dark lines represent mean and shaded region represents standard error assuming a negative binomial distribution of the data. (B–D) Estimated mean number of invertebrate taxa fished per country assuming different penalties for increased taxonomic precision. Dark blue line indicates estimate, light blue shaded region indicates standard error assuming a negative binomial distribution of the data, and the dark blue shaded regions indicate an estimated trend adjusted for increasing taxonomic precision in reporting. (B) Assumes each loss of an aggregated group results in 2 new species level designations, (C) assumes 3, and (D) assumes 4.
(0.24 MB TIF)

Figure S3 Percentage of all countries reporting catch of various invertebrate taxonomic and species groups. Dark lines represent smooth estimates obtained from a loess smoother (smoothing span 50% of the data). Light lines represent unfiltered data.
(0.28 MB TIF)

Figure S4 An example invertebrate catch series arranged by country for one invertebrate taxa: bivalves. Red lines indicate loess smoothed fits. Plots are ordered by cumulative catch since 1950. Vertical grey bars in title bars indicate log transformed cumulative catch, with bars near the right indicating the greatest cumulative catch and bars near the left indicating the least cumulative catch.
(0.69 MB TIF)

Figure S5 Illustration of our algorithm for dynamically assigning fishery status. Dots represent raw catch values, grey lines represent 3 of the loess functions fit to the data. Loess functions were built dynamically for each year but for clarity we show only the 3 functions which resulted in a change in status. By default a fishery was categorized as “expanding” until one of the following criteria was met: when there was at least 5 years since a maximum in the smoothed catch the fishery was classified as “fully exploited”, when smoothed catch fell below 50% of maximum smoothed catch the fishery was classified as “over-exploited”, and when smoothed catch fell below 90% of maximum catch the fishery was classified as “collapsed or closed”.
(0.17 MB TIF)

Figure S6 Percentage of fisheries for species from various functional groups that were categorized into the 4 fishery status categories. See section *Assessment of fishery status from catch trends* and Fig. 4C for a description of the how the species were assigned to the functional groups.
(0.18 MB TIF)

Figure S7 Demonstration of our fishery status assessment algorithm to simulated data. (A–C) Example of simulated increasing and then stationary catch series with multiplicative log-normal error about a random mean: log standard deviation of error of 0.10 (A), 0.25 (B), and 0.50 (C). Black lines indicate unfiltered catch. Red lines indicate loess smoothed fits. (D–F) Predicted stock status (expanding = green, fully exploited = yellow, over-exploited or restrictively managed = orange) from simulated data showing the robustness of our method to variability in the data as indicated for A, B, and C, respectively.
(0.25 MB TIF)

Figure S8 Characteristics of actual and simulated catch series. Frequency of log of mean catch values by fishery and log of the standard deviation of the residuals after fitting a loess smoother to each series (span = 0.5) from global invertebrate fisheries (A, B; grey background shading), and simulated series with σ = 0.1 (C, D), σ = 0.25 (E, F), and σ = 0.5 (G, H). See section *Verification of fishery status estimation using simulated data* for a description of σ. Red and blue vertical lines indicate median values.
(0.39 MB TIF)

Figure S9 Methods used to determine years of initial peaks in catch. (A) Illustration of our algorithm for assigning year of initial peak catch. Dots represent raw catch values, grey line represents loess function fit through the entire catch series, and red line indicates loess function fit through data up to the year of initial peak catch. A fishery was considered to have peaked if there was at least 500 tonnes of catch, at least a 10% decline from peak catch, and at least 3 years of data after the peak in catch. This algorithm was applied dynamically each year until the first instance of peak catch was observed. (B) Illustration of sampling time to peak for one censored fishery (Fishery A, red circle). Fisheries for which time to peak could be calculated are shown with solid dots in the shaded blue triangle. Censored fisheries for which time to peak was sampled are shown with open dots. Vertical dashed line indicates known year in which Fishery A surpassed 10% of its maximum observed catch. Fishery A could therefore have been assigned a time to peak from any value above 10 years, as indicated by a horizontal dashed line, and before 1970 (dark blue shaded region). This sampling was repeated 1000 times.
(0.25 MB TIF)

Figure S10 An example of time to peak catch vs. year of fishery initiation by taxonomic grouping for one random sampling of censored fisheries (red dots). Black dots represent known data points. In our analysis, the red dots were resampled 1000 times from possible time to peak values. Blue dots represent fisheries for which there were no fisheries to sample from. These were set to the maximum observed number of years for the earliest fishery affected (the left-most blue dot).
(0.34 MB TIF)

Table S1 Invertebrate catch for the 6 LMEs with the greatest total catch from 2000–2004. Also shown are the 3 taxonomic groups within each LME with the greatest catch. Catch values shown are annual averages over the 5-year span. LMEs are ordered by decreasing catch and within the LMEs the taxonomic groups are ordered by decreasing catch of that taxon.
(0.03 MB PDF)

Table S2 Distance and starting year of sea cucumber fisheries by country. Listed are each country's largest city (by population), its location, its distance from Hong Kong, the starting year of the sea cucumber fishery, and a verification reference.
(0.09 MB PDF)

Table S3 Major gear groupings of gear categories from the Sea Around Us Project catch database.
(0.03 MB PDF)

Table S4 Classification of invertebrate taxonomic groups into primary and secondary functional groups. Taxa are ordered approximately by decreasing trophic level.
(0.05 MB PDF)
