# Supplementary material for: Rapid Global Expansion of Invertebrate Fisheries: Trends, Drivers, and Ecosystem Effects
Source: PLoS One. 2011 Mar 8;6(3):e14735. doi: 10.1371/journal.pone.0014735 (PMC3050978; doi:10.1371/journal.pone.0014735)
Supplement: Text S1 — Supplementary description of the methods. (0.15 MB PDF) [file pone.0014735.s001.pdf]

Rapid Global Expansion of Invertebrate Fisheries: Trends, Drivers, and Ecosystem Effects
Supplementary Material: Text S1

Sean C. Anderson, Joanna Mills Flemming, Reg Watson, Heike K. Lotze

Temporal and spatial catch trends

Global catch data (i.e., reported landings) for all harvested invertebrate species were obtained from the Sea Around Us Project (http://seaaroundus.org) [1]. The data are based on landings reported to FAO, but have been quality checked and where possible, replaced with more precise versions from regional organizations such as the Northwest Atlantic Fisheries Organization (NAFO), the Commission for the Conservation of Antarctic Marine Living Resources (CCAMLR), and the International Council for the Exploration of the Sea (ICES) [2]. Known reporting errors, for example in Chinese records [3], are corrected as best possible. All such corrections are documented (see http://seaaroundus.org/doc/saup_manual.htm#13).

The Sea Around Us catch data are recorded by (i) which country reported the catch and (ii) the assumed LME in which the fishing was completed, for which catches are assigned to 30 x 30 minute cells using a series of rules taking into account where the catch was reported caught, known species' distributions, and fishing access agreements [1]. We mapped spatial patterns in global catches as the mean annual invertebrate catch per 100 km^2 in each LME from 2000–2004 (Fig. 1A) and by four taxonomic groups (Fig. S1). We also list the main LMEs and their main invertebrate catches in Table S1.

Temporal trends from 1950–2004 were derived for overall invertebrate catch, total finfish and invertebrate catch, and mean invertebrate catch per country per year (Fig. 1B). Confidence intervals were calculated under the common assumption that the catch data followed a log-normal distribution [4]. Trends were similar when we used the median instead. Wherever possible, we corroborated the observed trends with recent taxa-specific global reviews. These included sea cucumbers [5–7], sea urchins [8], squids [9], octopus and cuttlefishes [10], shrimps [11], gastropods [12], lobster, bivalve, and crab fisheries [13].

Estimating the legitimate increase in the diversity of species fished

To some extent, the increasing diversity of taxa reported in the Sea Around Us Project database is a function of the increasing taxonomic precision of reporting over time (Fig. 1C). For example, Malaysian

crustacean catch was recorded as Crustacea until 1986 before being split and reported as Sergestidae and *Panulirus*. There appeared to be a slowing or leveling of the mean number of species or group-level taxa reported per year since about 1980 (Fig. S2A). Therefore, we approximated the degree to which the increasing diversity reflected a true trend of an increasing number of species being targeted by fisheries.

As a first step, we excluded small fisheries because (i) we wished to focus on major fisheries and (ii) small fisheries were more likely to appear and disappear in the catch series (assuming some are experimental) thereby confusing the issue of diversity of fisheries. Thus, we included only those fisheries in which catch surpassed 1000 t/year since 1950 and which had at least 5 consecutive years of data. To exclude years in which a taxa or species was minimally fished, we excluded years in which a country reported catching less than 0.5 t of a taxonomic group or species.

We then flagged a fishery as potentially halting due to increased taxonomic precision if (i) the catch trend ended with over 1000 t/year before the end of the dataset (2004) and (ii) the taxonomic precision was broader than a species level designation (e.g., "Crustacea"). These were cases in which catch might have been reported for an aggregated group but was then reported in multiple more specific taxonomic divisions. We summed these instances cumulatively assuming that on average each instance resulted in a division from 1 broader category to 2, 3, or 4 specific categories (Fig. S2B–D). Each instance of a possible transition from an aggregated group to a species level designation division would have to result in at least 3 or 4 additional specific taxonomic divisions to affect the overall trend (Fig. S2B–D).

We note that this method does not account for instances where a country started reporting a fishery at a species level designation and continued to report that species in a group level designation. However, we see no method of discerning these instances on a global scale.

Taxonomic grouping

Globally, over 1200 taxonomic groups and species are reported caught in invertebrate or finfish fisheries, however, only the top species (based on cumulative catch since 1950) are recorded individually by the Sea Around Us Project with the remaining aggregated into groups such as "crustaceans" and "molluscs" (http://seaaroundus.org/doc/saup_manual.htm#8.6). Further, the Sea Around Us Project has aimed to disaggregate catch reported in aggregated taxonomic groups where possible, based primarily on taxonomic catch distribution in surrounding areas and known species' distributions, limiting the candidate taxa to

those reported by the same country in other years or by countries in the same LME (http://seaaroundus.org/doc/saup_manual.htm#8.4.5).

Thus, we obtained catch data for a total of 302 "taxa" (including 213 species). For our analyses, we looked at the number of taxa (species or species groups) fished over time (Fig. 1C), and catches for each of 4 aggregated taxonomic groups (crustaceans, bivalves, echinoderms, cephalopods), and 12 species groups (crabs, cuttlefishes, gastropods, krill, lobsters, octopus, sea cucumbers, sea stars, shrimps and prawns, squids, and urchins) (Fig. 2).

Increasing number of countries fishing

We extracted the number of countries reporting invertebrate catch from 1950–2004 as an indicator of the number of countries participating in invertebrate fisheries. One problem is that in the Sea Around Us Project database the designation of countries can change over time. For example, Samoa became independent from New Zealand in 1962 and appears independently in the data set from 1978 onwards. The overall classification of countries is not static. Such changes in the number of countries reporting catches over time are reflected in the overall number of countries reporting any catch for both finfish and invertebrate species. We have included this trend as a reference line (Fig. 1C, dashed red line).

Overall, the country designation variation was small compared to the much larger changes seen as a result of increasing participation in invertebrate fisheries. Nonetheless, we took this overall reporting trend into account and scaled the number of countries reporting catch of different invertebrate taxonomic and species groups to the total number of countries fishing finfish or invertebrates in any given year (Fig. S3).

Assessment of fishery status from catch trends

Although overall catch of invertebrate fisheries has been increasing, individual fisheries by taxa and country show a less optimistic picture (Fig. S4 for example). Previous attempts have been made to categorize the status of fisheries using catch data [13–16] as underdeveloped (prior to reaching 10% of maximum catch), expanding (prior to 50% of maximum catch), fully exploited (50% to 100% of maximum catch), over-exploited (decreased to 10% to 50% of maximum catch), and collapsed or closed (decreased to $<$ 10% of maximum catch). However, these approaches (i) can incorrectly categorize a fishery as over-exploited or collapsed due to single or multiple years of anomalous high catch and (ii) require all

non-declining fisheries to be categorized as fully-exploited by the end of the time series. Analysis of fishery status from catch trends will always remain an approximate science since catch can be affected by many variables other than stock status [17]. However, since catch is the only consistent metric we have for the vast majority of invertebrate fisheries, we developed a modified method for defining fishery status (Fig. S5) designed to take into account 2 shortcomings of the above technique.

(i) *An anomalous year of high reported catch could potentially induce false "collapses"* [18]. Given the variability in fisheries catches, even a stationary catch series will at some point exhibit a year of relatively high catch with subsequent years then categorized as over-exploited (or collapsed/closed). To reduce the effect of such anomalous high values, we filtered catch using a smoother that is robust to outliers — a loess smoother [19–21] from the function *loess* in the R statistical package [22] with a smoothing span of 0.5. This smooths the catch series, thereby down-weighting the impact of any outlying values. Such an approach is conservative in that it will require more evidence than a single high catch value before categorizing a fishery as over-exploited. We demonstrate the conservativeness of our approach using simulated data; see the subsequent section *Verification of fishery status estimation using simulated data.*

(ii) *Previous analyses categorized all fisheries that hadn't declined as fully developed by the end of the catch series.* This is likely untrue in the majority of cases, especially for newly emerging or expanding invertebrate fisheries that have not yet reached a peak and are still expanding. Therefore, if a catch series had not peaked within 5 years of its end, we categorized the fishery as expanding.

An important feature of our analysis was that we determined a fishery's current status based on only the data obtained up until that point. If alternatively we had used the entire catch series then we would have generated the false perception that more and more fisheries have become fully- or over-exploited in recent years. For example, what may have appeared to be a peak in catch after 10 years may not have appeared so if we had observed and smoothed the data over an additional 30 years; see the section *Analysis of fishery development time.* Essentially our approach enabled us to treat old and recent fisheries equally as they developed.

We note that our method necessitated a different definition of "fully exploited". Previously [13–16], a retrospective approach was taken and considered a fishery fully exploited if the catch was anywhere above 50% of the maximum catch. With our dynamic approach, we defined fully exploited as anywhere after a peak in catch and before catch fell below 50% of that peak (Fig. S5). Additionally, a fishery with less than 10 years of data at any time step was not evaluated (indicated in Fig. 3A as light green).

Verification of fishery status estimation using simulated data

Since a criticism of previous fishery status estimation approaches has been the incorrect finding of an increasing number of collapses due to data variability or anomalous years of catch [18], we demonstrate the robustness of our method to assigning false collapses or declines using simulated data. Our simulated catch series (C_t) last 55 years (T). They start at zero tonnes in the first year and increase according to the first quarter of a sine wave before leveling off at a maximum catch value (C_m) randomly selected from a log-normal distribution with a median value of 5000 t. The period of the wave, i.e., the time to maximum development of the fishery (d_t), was randomly selected from a uniform distribution varying between zero and 30 years based on the approximate ranges observed in the Sea Around Us Project's catch data for invertebrate fisheries. We added varying levels of multiplicative log-normally distributed random noise (z_t) (with a first-order autocorrelation of 0.2) to the simulated catch trends (Fig. S7A–C):

$$z_t = LogN(0, \sigma^2)$$

$$C_t = \begin{cases} \sin(\pi t/2d_t) \cdot C_m \cdot z_t & \text{if } t = 0 \ldots d_t \\ C_m \cdot z_t & \text{if } t = d_{t+1} \ldots T \end{cases}$$

We demonstrate our method applied to data with log standard deviations of 0.10, 0.25, and 0.50 (Fig. S7D–F). At each of these 3 levels of variation we ran our simulation 1000 times and found the false positive rate (categorizing a fishery as "over-exploited" or "collapsed" when it should be "expanding" or "fully exploited") low at 0% for 0.01, 2% for 0.25, and 24% for 0.50 log standard deviation. We note that the variation in Fig S7D exceeds the variation seen in the Sea Around Us Project catch database for invertebrates (Fig. S8). Further, the autocorrelation of z_t exceeds that in the invertebrate catch series: the median first-order autocorrelation of residuals from loess smoother fit to actual catch was 0.05. Therefore, the false positive rate due to anomalous values in our simulated data should exceed that in the Sea Around Us Project's catch data.

Correlation of distance from Hong Kong with fishery initiation year

When a resource becomes locally depleted, fisheries often respond by expanding the fishing area. On a global scale, this could mean that if one country has depleted its resource, other countries may start

fishing it. Over time, the resource is fished further and further away from its original country or countries. Such spatial expansion and depletion has been suggested for global sea urchin fisheries [23]. We were interested in whether other invertebrate fisheries followed this trend. Few species, however, have a single strong market, making such detection difficult. We chose to investigate sea cucumbers because they have one strong market in Asia. Additionally we investigated squids, which have 3 main markets [24, 25], but we were unable to locate historical import statistics for squid fisheries of sufficient length for all major importing nations.

For sea cucumbers, the majority of catch (64% of the cumulative import volume since 1950 [25]) is imported by Hong Kong where it is processed before most of it is then directed to China [6, 26]. The vast majority of the remaining import volume is imported by nearby Asian countries [25]. We reasoned that great circle distance could be used as a proxy for the spatial distance, and therefore ease of transportation, between the exporting and importing nations.

For each country, we determined the great circle distance between its city with the largest population (as a proxy for the city with the largest cargo airport) and the main Hong Kong freight operator, Hong Kong Air Cargo Terminals, at Hong Kong International Airport (Table S2), which handles over 70% of Hong Kong's air cargo [27]. City population data (as of January 2006) and latitude and longitude were obtained from the dataset *world.cities*, which is part of the R [22] package *maps* [28]. Although the largest cargo airport may not always be found in the largest city by population, most countries are small enough geographically (compared to their distance from Hong Kong) to not affect our results. In the case of the United States and Canada, however, east and west coast regions started fishing at different times, and, due to the width of the continent, are of substantially differing distances from Hong Kong. Here we used the coordinates of the largest city (by population) in each Canadian region (west and east coast) or US state as the assumed location of the largest air cargo airport. We natural log transformed the distance data for both ease of visual interpretation and normality of the linear regression residuals.

To determine a starting year for each fishery (Table S2) we calculated the year at which catch (smoothed via a loess curve as outlined earlier) passed 10% of its first peak in catch; see subsequent section *Analysis of fishery development time*. For the east and west coast Canadian fisheries, catch trends and 10% starting years were calculated based on governmental reports [29–32]. For the United States, where separate catch trends were unavailable, we used the reported years of directed fishery initiation from the literature [33, 34].

We note that this analysis is an extension of an analysis conducted in Anderson et al. [35]. Here, we used 10% of the first peak in catch — dynamically fitting a loess smoother each year as in the *Assessment of fishery status from catch trends* and *Analysis of fishery development time* sections — as opposed to 10% of the overall peak in catch; we used reported starting years as opposed to empirically derived starting years for the United States fisheries; and we used a linear regression of log-transformed catch data as opposed to a generalized linear model.

Analysis of fishery development time

We were interested in whether there was evidence that newer fisheries were developing more rapidly than in the past. We assessed this by checking for a relationship between when invertebrate fisheries began and the time when they achieved their first peak in catch ("initial peak catch"). We used a meta-analysis of the correlation coefficients of these two variables. We describe this analysis in detail in the following paragraphs.

Here, a fishery was defined as 1 of the 12 larger taxonomic groupings (Fig. 3C) as reported by an individual country. We excluded sea stars and krill due to the limited number of countries with substantial fisheries (leaving 10 fisheries). To focus on substantial fisheries, we discarded all fisheries that didn't surpass 1000 t/year. We made an exception for the lower volume sea urchin and sea cucumber fisheries for which we took a minimum catch of 250 t. Our overall conclusions were invariant to choices of cutoffs from 500–2000 t (for the higher volume fisheries).

Catch trajectories can have multiple smaller local peaks together with an overall peak. For example, Fig. S4 shows world bivalve fisheries by country. If we naively calculated the peak catch from the entire available catch trajectory we would be more likely to be measuring local peaks (rather than overall peaks) with fisheries that started more recently. This alone would falsely generate the trend for which we were testing. To avoid this time based bias we calculated the time it took for each fishery to develop to the first peak in catch.

For each year, a loess curve was fit to the data (as outlined earlier). A fishery was only evaluated if there were at least 5 years of data to ensure there would be enough data to conclude a peak had occurred. Fisheries with less than 5 years of data were considered censored.

For each year, the smoothed catch trajectory was built and catch was considered to have reached initial peak catch if (Fig. S9A):

1. a maximum in catch occurred and was not within 3 years of the end of the catch series at that step (so we had enough subsequent data to ensure a peak),

2. a maximum in catch was at least half of our cutoff for considering the fishery — 500 t for most taxa and 125 t for sea cucumbers and sea urchins (to avoid small peaks during the variable catch portion at the start of the fishery), and

3. a maximum in catch was at least 10% greater than the catch at the end of the catch series at that step (to ensure a peak and not a stationary catch series).

If even one of these criteria was not met, then our knowledge of peak catch for that fishery was considered censored as of that year.

We considered the year in which smoothed catch surpassed 10% of the smoothed peak catch as the starting year. This approximates when the fishery became a substantial directed fishery. If a fishery was censored then we took 10% of the maximum observed smoothed catch as the initiation year. We removed all fisheries that began at greater than 10% of the maximum catch (i.e., fisheries that began prior to 1950). This simplified our analysis and allowed us to make inferences for fisheries that began between 1950 and 2000.

Central to this analysis, we had to deal with the censored fisheries that had yet to achieve peak catch. The possible range of censored fishery time to peak catch values increases over time — it could be anywhere in a missing triangle above the known data (Fig. S9B).

To account for these censored fisheries we assumed the null hypothesis that there had been no change in the distribution of times to peak for recent fisheries compared to fisheries that began between 1950 and 1970 (Fig. S9B). For fisheries in which there was no precedence (fisheries that had lasted longer than any other fishery in that taxa and still had not peaked), we assigned the maximum observed time for that taxa (Fig. S10). We chose this approach to be most conservative. If we had assigned the maximum length for which we had observed each fishery as the time to peak we would have had more slower developing older fisheries. This would have created a linear downward trend in time to peak — the trend we were testing for. We proceeded to determine if we could still detect a pattern between year of initiation and time to the first peak in catch despite assigning simulated values to the censored fisheries (Fig. S10).

We compared the correlation between year of initiation and year of first peak in catch within each taxonomic group. We repeated our correlation analysis 1000 times, each time resampling the censored

fisheries. This approach generates 2 kinds of uncertainty in our correlation estimates: uncertainty due to the resampling of censored values ("missingness") and uncertainty on each individual correlation coefficient. For each taxa, we derived the combined standard error by taking the median of the individual standard errors. We show the median correlation coefficients and 95% confidence intervals (Fig. 3C). We combined the median correlation coefficients using inverse-variance weighted meta-analysis [36]. We estimated a change in time to peak between 1960 and 1990 by repeating our analysis with slope estimates (instead of correlation coefficients) and using the meta-analytic slope estimate to predict on the year scale. The approximate range of possible time to peak values (± 3 years) was based on a 95% confidence interval.

Our overall results were robust to both our choice of peak catch algorithm and smoothing function. We tested our analysis with loess functions with smoothing spans ranging from 0.25 to 0.9 and with running medians of length 3 through 9. Finally, the overall trend remained when we tested our analysis substituting robust regression (iterated re-weighted least squares using MM-estimation [37, 38]) for least squares regression.

Potential habitat impacts

To assess the potential habitat effects of different invertebrate fisheries, we calculated the total invertebrate catch and the number of taxa fished by different gear types (Fig. 4A,B). The Sea Around Us Project derived gear associations for taxonomic groups primarily from books, journals, and Internet sources [39]. Where unavailable, gear associations were interpolated based on the type of organism, country fished, and FAO area where the gear was used [39].

There were 19 types of fishing gear recorded for invertebrates, which we grouped into 6 broader groups based on their potential habitat impact (Table S3). Hand dredges or rakes can have short term effects (up to a year) on marine habitat and its associated community but these effects are unlikely to remain on longer time scales unless long lived species are present [40, 41]. Lines and hooks represent a substantive bycatch concern for threatened sea turtle [42, 43] and seabird populations [44] among other taxa. Diving and grasping likely has the least impact on habitat and bycatch but is used in a small proportion of the fisheries by taxa and especially by volume (Fig. 4A,B). Traps and pots are unlikely to have significant marine habitat impacts [45] although present an issue of marine mammal entanglement [46] and bycatch [47]. Nets and midwater trawls, while avoiding the benthic habitat damage of benthic

trawling and dredging have substantial bycatch issues with taxa such as cetaceans [48], sea turtles [49], and sharks [50]. Benthic trawling and dredging, which combined comprised 53% of invertebrate catch by volume and 71% of the species or species groups fished, can have great impact on benthic habitat and communities; see *Results and Discussion* in the main text [51, 52].

Functional group analysis

To evaluate the potential food-web and ecosystem impacts of different invertebrate fisheries, we assigned functional groups to larger taxonomic groupings (Fig. 4C). Functional groups were assigned as primary or secondary roles within that functional group according to the primary literature and reference books (Table S4). The "habitat" group included species known to form habitat themselves. Therefore, we excluded species such as sea urchins, which can influence marine habitat by predating on kelp. Trophic levels were obtained from the Sea Around Us Project (http://www.seaaroundus.org/doc/saup_manual.htm#12). They were mainly derived from Froese and Pauly [53].

In order to quantify the ecosystem effect, we extracted the overall removal (catch) of each primary functional group. Based on Fig. 4C, we amassed the total catch per functional group averaged over 2000–2004 (the 5 most recent years available) (Fig. 4D). This does not include renewal of resources via recruitment and re-growth.

Estimation of bivalve filtering capacity

We estimated the consequence of removing filter feeding bivalves from the ocean, in terms of their capacity to filter water, using filtration rates reported in the literature [54–56]. Newell [54] estimated the filtering capacity of American oysters (*Crassostrea virginica*) in Chesapeake Bay (US) to be 5 $L \cdot g^{-1} \cdot h^{-1}$. We applied this value to the mean global catch of bivalves for the last 5 years of our data (2000–2004, 2.72 million t) to estimate the removal of filtering capacity per year. We converted wet weight landings to shell-free dry weight by the median value reported in the literature for all bivalves (8.6% of wet weight) as reported by Ricciardi and Bourget [56]. We converted these values into Olympic sized swimming pools for comparison. We estimated the volume of a pool as $2.5 \cdot 10^6$ l, pool volume = 50m · 25m · 2m.

Newell's estimate of filtration capacity [54] was made for one bivalve species in one geographic region. Therefore, we checked our results using filtration rates compiled by Ricciardi and Bourget [56], which were obtained under ideal laboratory conditions and should typically represent similar values to what would

be observed in nature across a range of bivalve species [55]. We used the first quartile, median, and third quartile values of the weight-based filtration rates (F) reported by Riisgard: $F = 6.47W^{0.72}$, where W is the shell-free dry weight. To simplify the analysis, we assumed an individual bivalve to be on average 1 g shell-free dry weight or ∼11.6 g wet weight [56]. Under these assumptions, with the three filtration rates we calculated the loss of filtration capacity to be ∼14.5 million pools per day ($\sim 3.6 \cdot 10^{10}\ m^3$; first and third quartile values: 12.2 and 17.5 million pools per day), a similar result to our estimate using Newell's approximation. We report the more conservative estimate in the *Results and Discussion* section in the main text.

Supporting References

1. Watson R, Alder J, Kitchingman A, Pauly D (2005) Catching some needed attention. Mar Pol 29: 281–284.

2. Zeller D, Pauly D, editors (2007) Reconstruction of marine fisheries catches for key countries and regions (1950-2005). Fisheries Centre Research Reports. Vol. 15 Issue 2.

3. Watson R, Pauly D (2001) Systematic distortions in world fisheries catch trends. Nature 414: 534-536.

4. Haddon M (2001) Modelling and quantitative methods in fisheries. FL, USA: Chapman & Hall/CRC.

5. Conand C (2004) Present status of world sea cucumber resources and utilisation: an international overview. In: Lovatelli A, Conand C, Purcell SW, Uthicke S, Hamel JF, et al., editors, Advances in sea cucumber aquaculture and management. FAO Fisheries Technical Paper 463, Rome, Italy: Food and Agriculture Organization of the United Nations. pp. 13–23.

6. Toral-Granda V, Lovatelli A, Vasconcellos M, Conand C, Hamel JF, et al. (2008) Sea cucumbers. a global review on fishery and trade. SPC Beche-de-mer Information Bulletin 28: 4–13.

7. FAO (2008) Sea cucumbers: A global review of fisheries and trade. Technical Report 516, Food and Agriculture Organization of the United Nations, Rome, Italy.

8. Andrew NL, Agatsuma Y, Ballesteros E, Bazhin E, Creaser EP, et al. (2002) Status and management of world sea urchin fisheries. Oceanogr Mar Biol Annu Rev 40: 343–425.

9. Payne A, Agnew D, Pierce G (2006) Trends and assessment of cephalopod fisheries — foreword. Fish Res 78: 1–3.

10. Boyle PR, Rodhouse P (2005) Cephalopods: Ecology and Fisheries. Wiley-Blackwell.

11. FAO (2008) Global study of shrimp fisheries. Technical report, Food and Agriculture Organization of the United Nations, Rome, Italy.

12. Leiva G, Castilla J (2002) A review of the world marine gastropod fishery: evolution of catches, management and the Chilean experience. Rev Fish Biol Fisheries 11: 283–300.

13. FAO (2009) The state of world fisheries and aquaculture 2008. Technical report, Food and Agriculture Organization of the United Nations, Rome, Italy.

14. Froese R, Kesner-Reyes K (2002) Impact of fishing on the abundance of marine species. ICES Council Meeting Report CM 2002/L:12: 1–16.

15. Sumaila UR, Khan A, Watson R, Munro G, Zeller D, et al. (2007) The World Trade Organization and global fisheries sustainability. Fish Res 88: 1–4.

16. Pauly D (2007) The Sea Around Us Project: Documenting and communicating global fisheries impacts on marine ecosystems. Ambio 36: 290-295.

17. Harley S, Myers RA, Dunn A (2001) Is catch-per-unit-effort proportional to abundance? Can J Fish Aquat Sci 58: 1760–1772.

18. Branch T (2008) Not all fisheries will be collapsed in 2048. Mar Pol 32: 38–39.

19. Cleveland WS (1979) Robust locally weighted regression and smoothing scatterplots. J Am Stat Assoc 74: 829–836.

20. Cleveland WS, Devlin SJ (1988) Locally weighted regression: An approach to regression analysis by local fitting. J Am Stat Assoc 83: 596–610.

21. Cleveland WS, Grosse E, Shyu WM (1992) Local regression models. In: Chambers JM, Hastie TJ, editors, Statistical Models in S, Wadsworth & Brooks/Cole, chapter 8. pp. 309–376.

22. R Development Core Team (2009) R: a language and environment for statistical computing. R Foundation for Statistical Computing, Vienna, Austria. URL http://www.R-project.org. ISBN 3-900051-07-0.

23. Berkes F, Hughes TP, Steneck RS, Wilson JA, Bellwood DR, et al. (2006) Globalization, roving bandits, and marine resources. Science 311: 1557–1558.

24. Sonu SC (1989) Export opportunities for U.S. squid. NOAA Technical Memorandum NMFS SWR-022, National Oceanic and Atmospheric Administration National Marine Fisheries Service, Southwest Region.

25. FAO (2009). Fisheries commodities production and trade: Fisheries and Agriculture Organization of the United Nations. Electronic database. http://www.fao.org/fishery/statistics/software/fishstat/en.

26. Jaquemet S, Conand C (1999) The beche-de-mer trade in 1995/1996 and an assessment of exchanges between the main world markets. SPC Beche-de mer Information Bulletin 12: 11–14.

27. Hong Kong General Chamber of Commerce (2009). Hong Kong business directory: Hong Kong Air Cargo Terminals Ltd. http://www.chamber.org.hk/.

28. Becker RA, Wilks AR, Brownrigg R, Minka TP (2009) maps: Draw geographical maps. R package version 2.1-0.

29. DFO (1996) Scotian Shelf sea cucumber. Technical report, Fisheries and Oceans Canada, 96/125E (Dartmouth, NS).

30. DFO (2002) Giant red sea cucumber. Technical Report C6-10(2002), Fisheries and Oceans Canada, Nanaimo, BC.

31. Hand CM, Hajas W, Duprey N, Lochead J, Deault J, et al. (2008) An evaluation of fishery and research data collected during the Phase 1 sea cucumber fishery in British Columbia, 1998 to 2007. Canadian Science Advisory Secretariat Research Document 065, Fisheries and Oceans Canada, Nanaimo, BC, Canada.

32. Rowe S, Comeau P, Singh R, Coffen-Smout S, Lundy M, et al. (2009) Assessment of the exploratory fishery for sea cucumber (*Cucumaria frondosa*) in southwest New Brunswick. Canadian Science

Advisory Secretariat Research Document 005, Fisheries and Oceans Canada, Dartmouth, NS, Canada.

33. Bruckner AW (2005) The recent status of sea cucumber fisheries in the continental United States of America. SPC Beche-de-mer Information Bulletin 22: 39–46.

34. Therkildsen N, Petersen C (2006) A review of the emerging fishery for the sea cucumber *Cucumaria frondosa*: Biology, policy, and future prospects. SPC Beche-de-mer Information Bulletin 23: 16–25.

35. Anderson SC, Flemming JM, Watson R, Lotze HK (2010) Serial exploitation of global sea cucumber fisheries. Fish Fish In press.

36. Cooper H, Hedges LV (1994) The Handbook of Research Synthesis. New York: Russel Sage Foundation.

37. Huber PJ (1981) Robust Statistics. New York: Wiley.

38. Venables WN, Ripley BD (2002) Modern Applied Statistics with S. Springer, 4th edition.

39. Watson R, Revenga C, Kura Y (2006) Fishing gear associated with global marine catches I. Database development. Fish Res 79: 97–102.

40. Kaiser M, Broad G, Hall S (2001) Disturbance of intertidal soft-sediment benthic communities by cockle hand raking. J Sea Res 45: 119–130.

41. MacKenzie C, Pikanowski R (2004) Gear effects on marine habitats: Harvesting northern quahogs in a shallow sandy bed at two levels of intensity with a short rake. N Am J Fish Manag 24: 1221–1227.

42. Lewison R, Freeman S, Crowder L (2004) Quantifying the effects of fisheries on threatened species: the impact of pelagic longlines on loggerhead and leatherback sea turtles. Ecol Let 7: 221–231.

43. Lewison RL, Crowder LB (2007) Putting longline bycatch of sea turtles into perspective. Conserv Biol 21: 79–86.

44. Lewison RL, Crowder LB (2003) Estimating fishery bycatch and effects on a vulnerable seabird population. Ecol Appl 13: 743–753.

45. Eno N, MacDonald D, Kinnear J, Amos S, Chapman C, et al. (2001) Effects of crustacean traps on benthic fauna. ICES J Mar Sci 58: 11–20.

46. Johnson A, Salvador G, Kenney J, Robbins J, Landry S, et al. (2005) Fishing gear involved in entanglements of right and humpback whales. Mar Mamm Sci 21: 635–645.

47. Johnson AE (2010) Reducing bycatch in coral reef trap fisheries: escape gaps as a step towards sustainability. Mar Ecol Prog Ser 415: 201–209.

48. Fertl D, Leatherwood S (1997) Cetacean interactions with trawls: A preliminary review. J Northw Atl Fish Sci 22: 219–248.

49. Crowder L, Crouse D, Heppell S, Martin T (1994) Predicting the impact of turtle excluder devices on loggerhead sea turtle populations. Ecol Appl 4: 437–445.

50. Stevens JD, Bonfil R, Dulvy NK, Walker PA (2000) The effects of fishing on sharks, rays, and chimaeras (chondrichthyans), and the implications for marine ecosystems. ICES J Mar Sci 57: 476–494.

51. Hiddink J, Jennings S, Kaiser M, Queiros A, Duplisea D, et al. (2006) Cumulative impacts of seabed trawl disturbance on benthic biomass, production, and species richness in different habitats. Can J Fish Aquat Sci 63: 721–736.

52. Tillin H, Hiddink J, Jennings S, Kaiser M (2006) Chronic bottom trawling alters the functional composition of benthic invertebrate communities on a sea-basin scale. Mar Ecol Prog Ser 318: 31–45.

53. Froese R, Pauly D, editors (2000). Fishbase 2000: concepts, design and data sources. ICLARM, Los Baños, Philippines.

54. Newell RIE (1988) Ecological changes in Chesapeake Bay: Are they the result of overharvesting the American oyster, *Crassostrea virginica*? In: Understanding the Estuary: Advances in Chesapeake Bay Research. Baltimore, Maryland: Chesapeake Research Consortium Publication, pp. 29–31.

55. Riisgard H (2001) On measurement of filtration rates in bivalves — the stony road to reliable data: review and interpretation. Mar Ecol Prog Ser 211: 275–291.

56. Ricciardi A, Bourget E (1998) Weight-to-weight conversion factors for marine benthic macroinvertebrates. Mar Ecol Prog Ser 163: 245–251.
