# Supplementary figures and images for: Rapid Global Expansion of Invertebrate Fisheries: Trends, Drivers, and Ecosystem Effects
Source: PLoS One. 2011 Mar 8;6(3):e14735. doi: 10.1371/journal.pone.0014735 (PMC3050978; doi:10.1371/journal.pone.0014735)

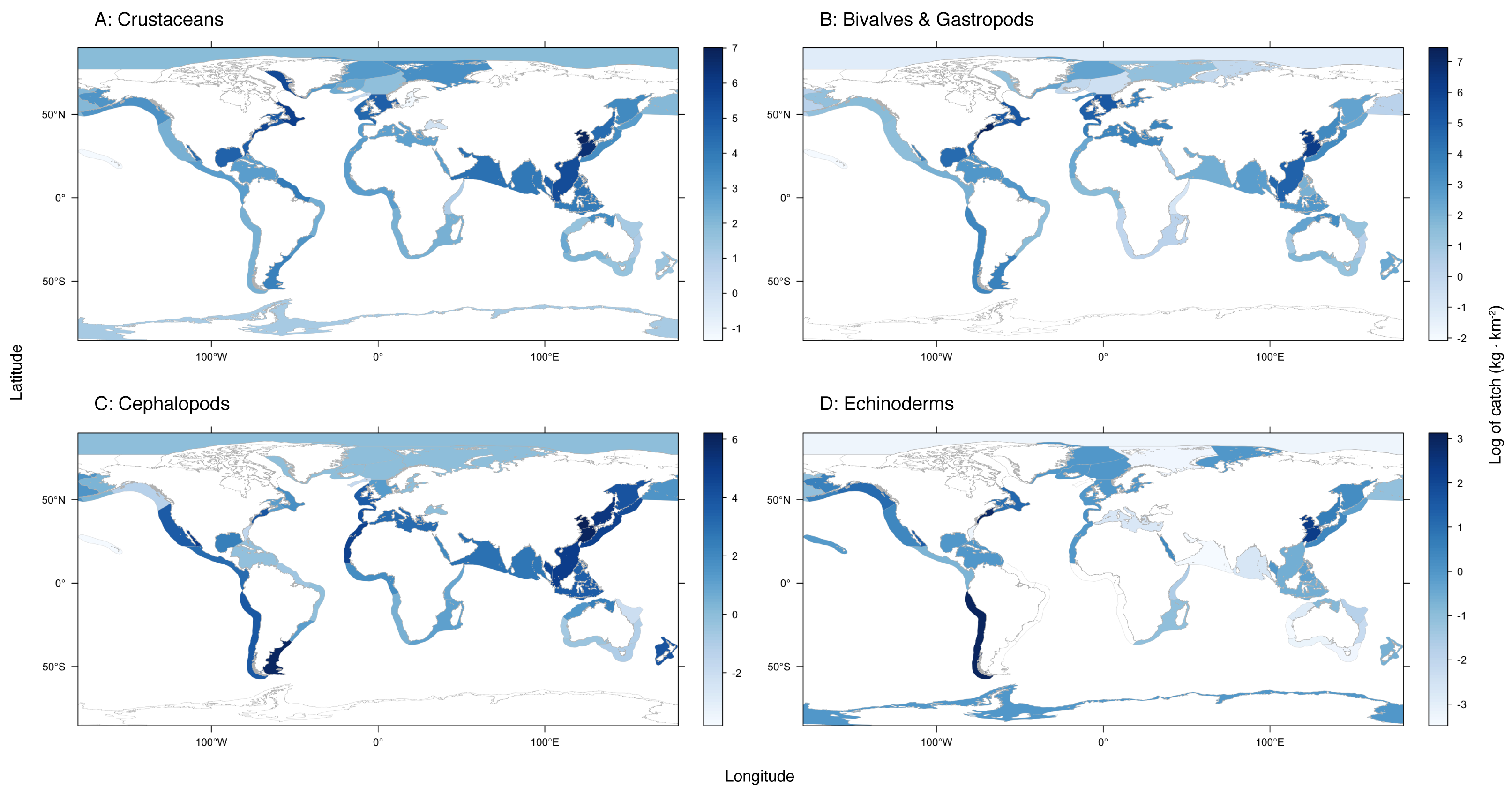

Supplement: Figure S1 — Mean annual invertebrate catch by taxonomic group in each Large Marine Ecosystem (LME) from 2000–2004. (1.13 MB TIF) [file pone.0014735.s002.tif]

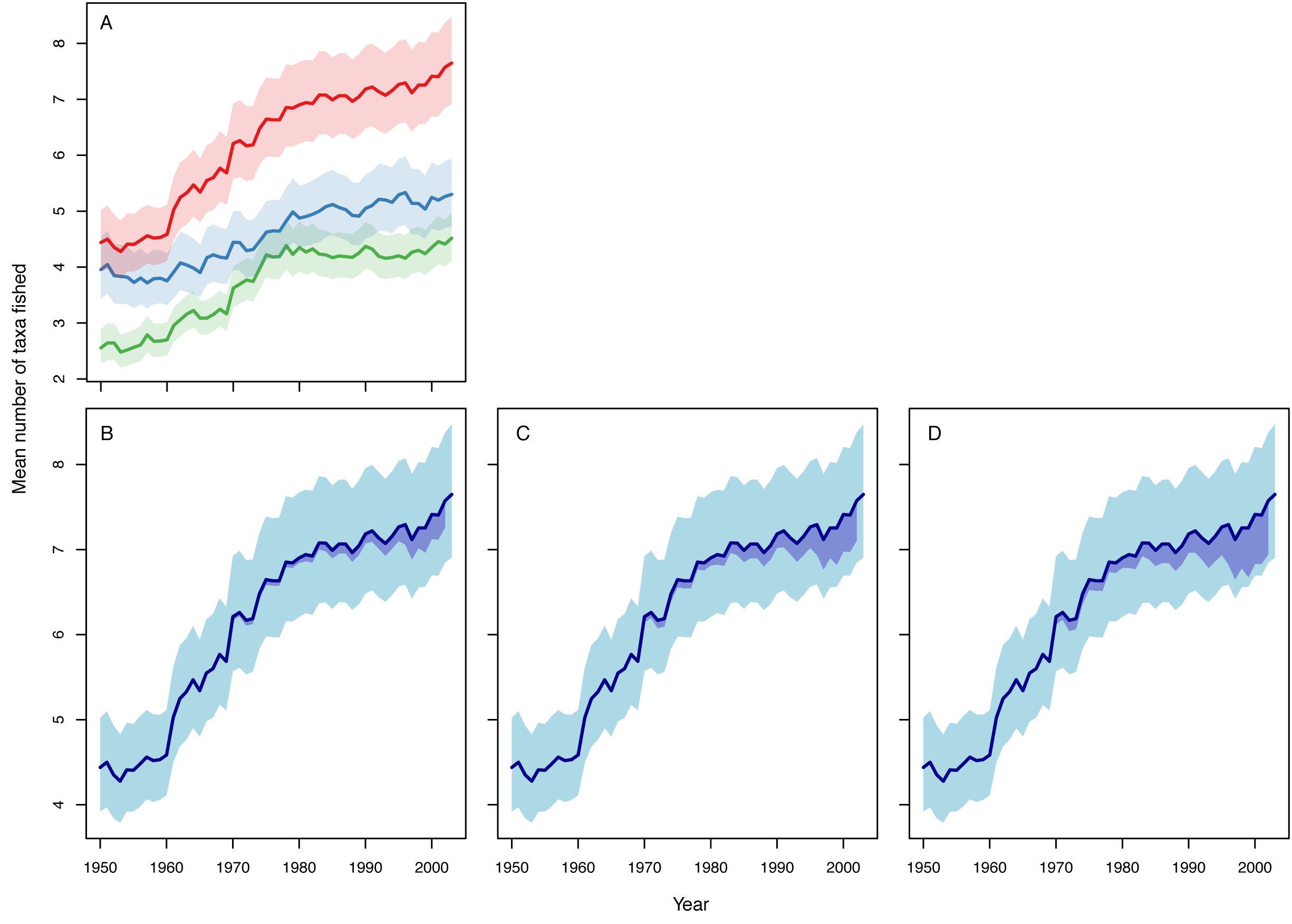

Supplement: Figure S2 — Effects of taxonomic precision in reporting on predicted trends in diversity of invertebrates fished. (A) Increasing reporting of invertebrate taxa fished divided into species level (blue), larger grouping level (green), and combined (red). Dark lines represent mean and shaded region represents standard error assuming a negative binomial distribution of the data. (B–D) Estimated mean number of invertebrate taxa fished per country assuming different penalties for increased taxonomic precision. Dark blue line indicates estimate, light blue shaded region indicates standard error assuming a negative binomial distribution of the data, and the dark blue shaded regions indicate an estimated trend adjusted for increasing taxonomic precision in reporting. (B) Assumes each loss of an aggregated group results in 2 new species level designations, (C) assumes 3, and (D) assumes 4. (0.24 MB TIF) [file pone.0014735.s003.tif]

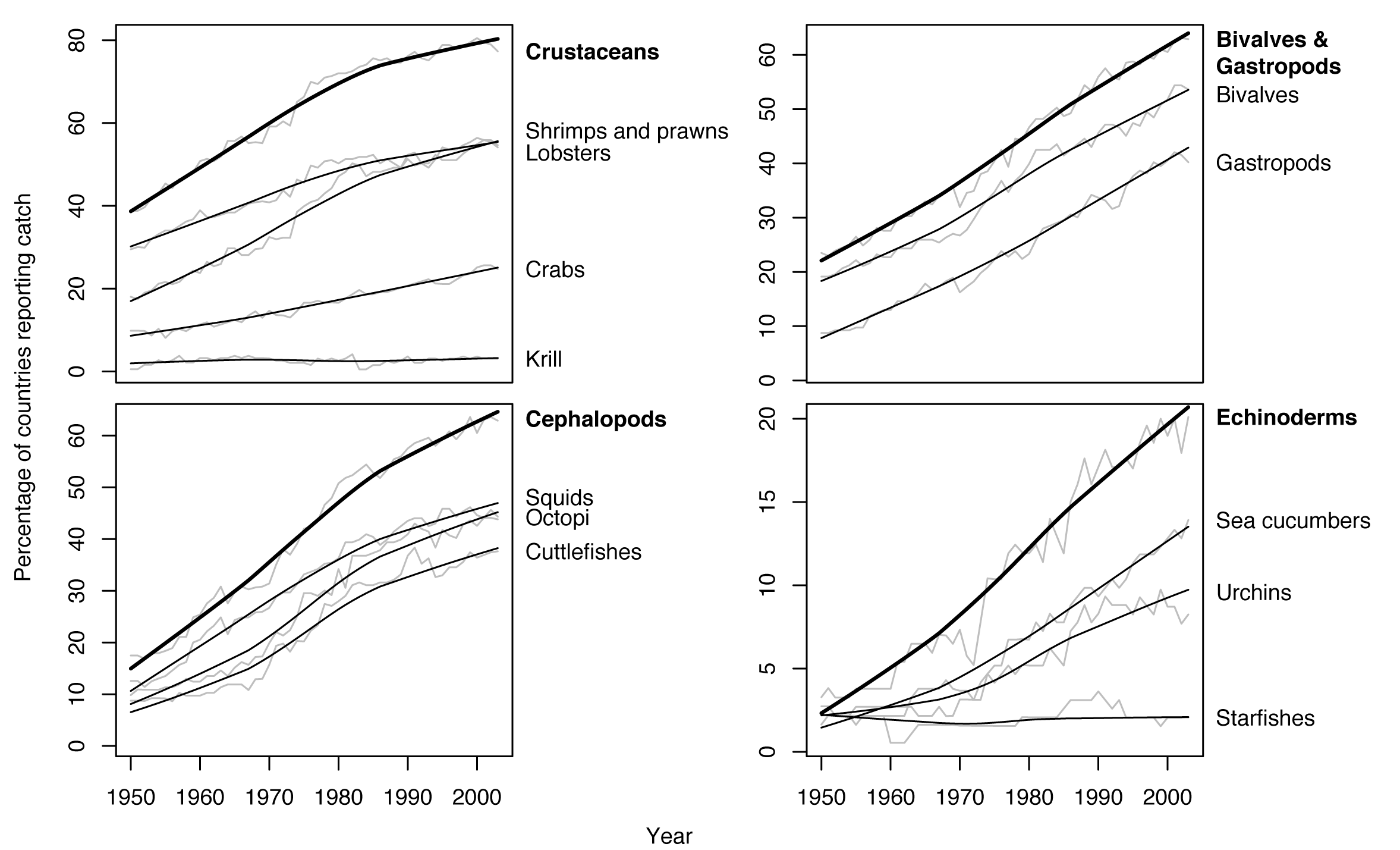

Supplement: Figure S3 — Percentage of all countries reporting catch of various invertebrate taxonomic and species groups. Dark lines represent smooth estimates obtained from a loess smoother (smoothing span 50% of the data). Light lines represent unfiltered data. (0.28 MB TIF) [file pone.0014735.s004.tif]

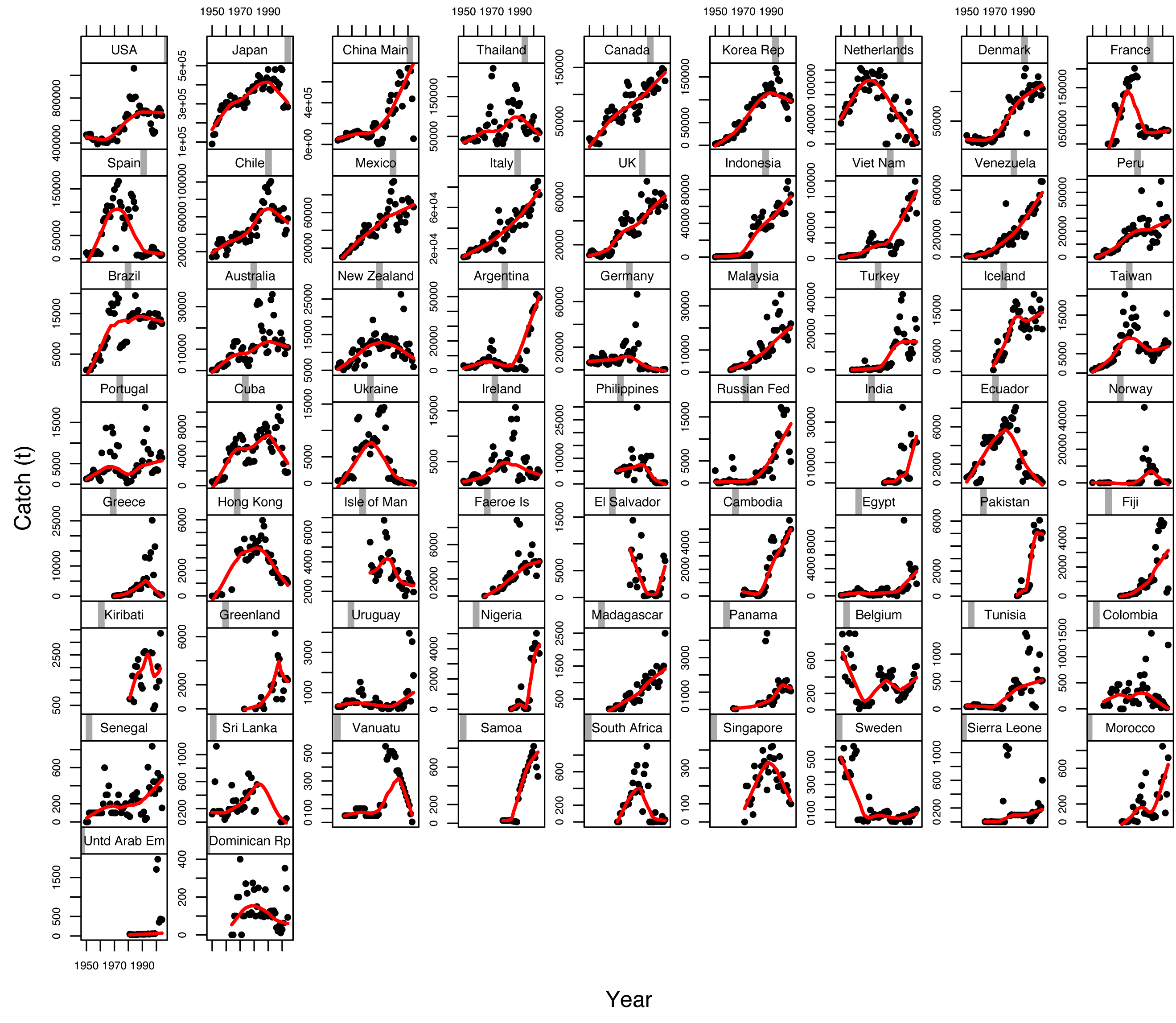

Supplement: Figure S4 — An example invertebrate catch series arranged by country for one invertebrate taxa: bivalves. Red lines indicate loess smoothed fits. Plots are ordered by cumulative catch since 1950. Vertical grey bars in title bars indicate log transformed cumulative catch, with bars near the right indicating the greatest cumulative catch and bars near the left indicating the least cumulative catch. (0.69 MB TIF) [file pone.0014735.s005.tif]

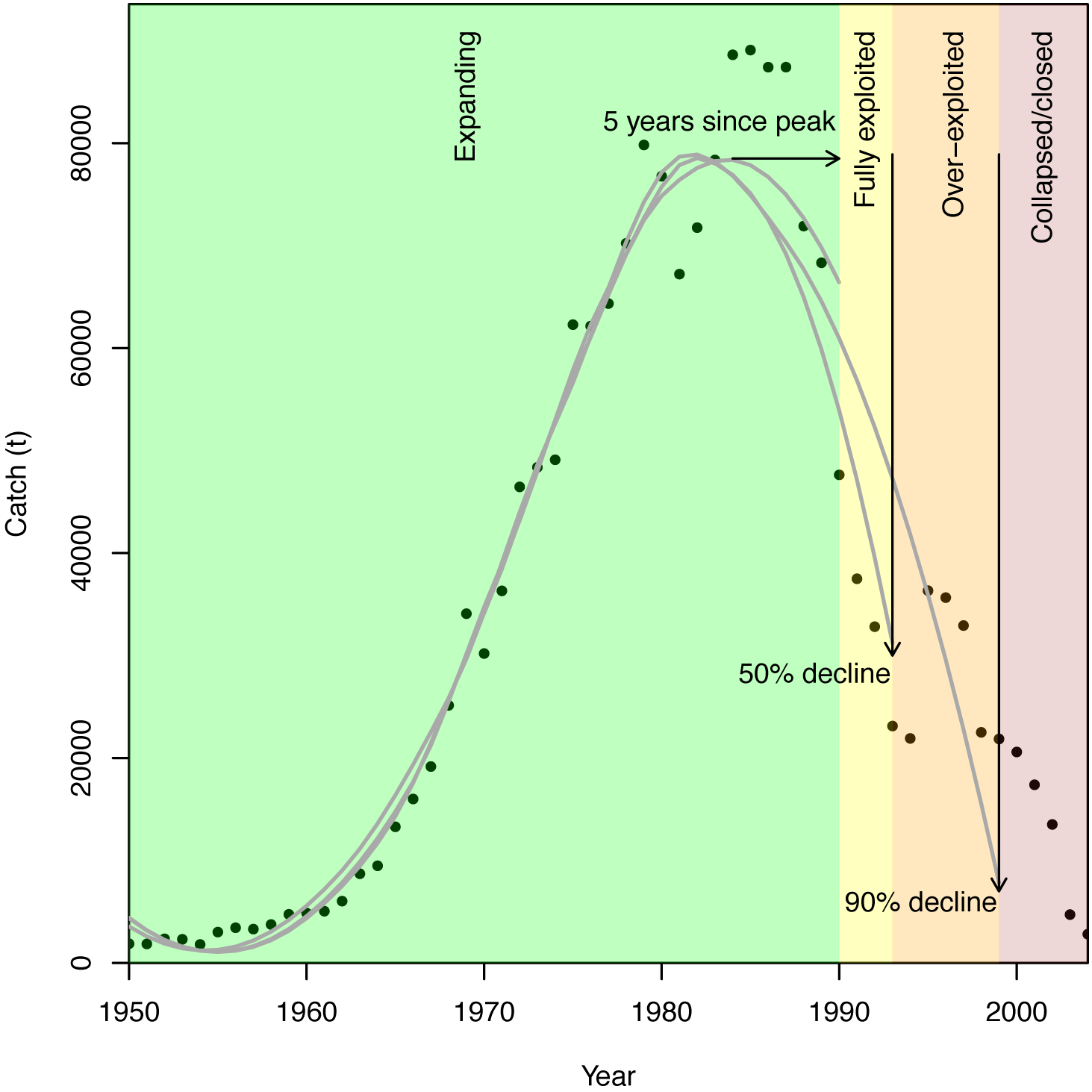

Supplement: Figure S5 — Illustration of our algorithm for dynamically assigning fishery status. Dots represent raw catch values, grey lines represent 3 of the loess functions fit to the data. Loess functions were built dynamically for each year but for clarity we show only the 3 functions which resulted in a change in status. By default a fishery was categorized as “expanding” until one of the following criteria was met: when there was at least 5 years since a maximum in the smoothed catch the fishery was classified as “fully exploited”, when smoothed catch fell below 50% of maximum smoothed catch the fishery was classified as “over-exploited”, and when smoothed catch fell below 90% of maximum catch the fishery was classified as “collapsed or closed”. (0.17 MB TIF) [file pone.0014735.s006.tif]

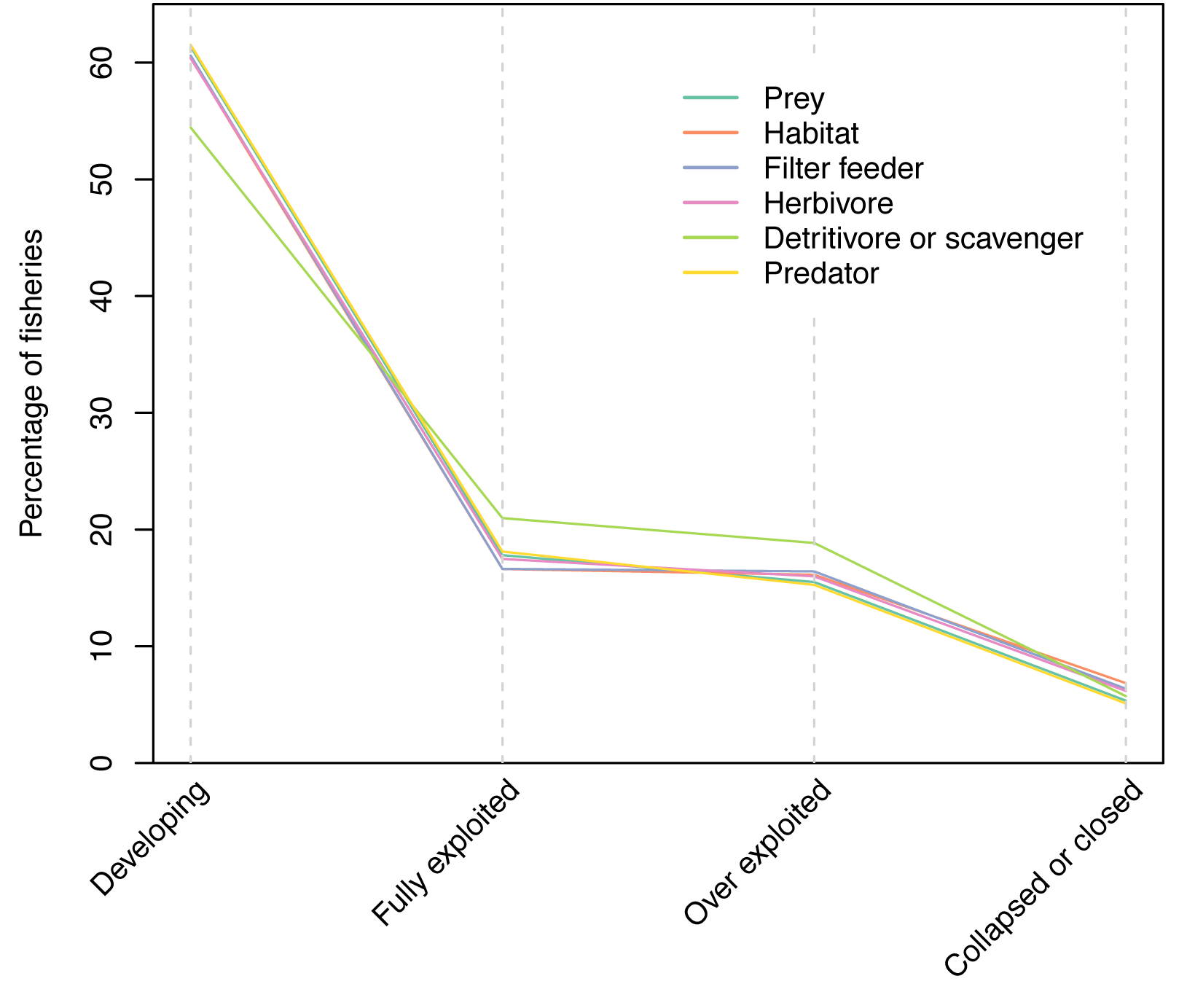

Supplement: Figure S6 — Percentage of fisheries for species from various functional groups that were categorized into the 4 fishery status categories. See section Assessment of fishery status from catch trends and Fig. 4C for a description of the how the species were assigned to the functional groups. (0.18 MB TIF) [file pone.0014735.s007.tif]

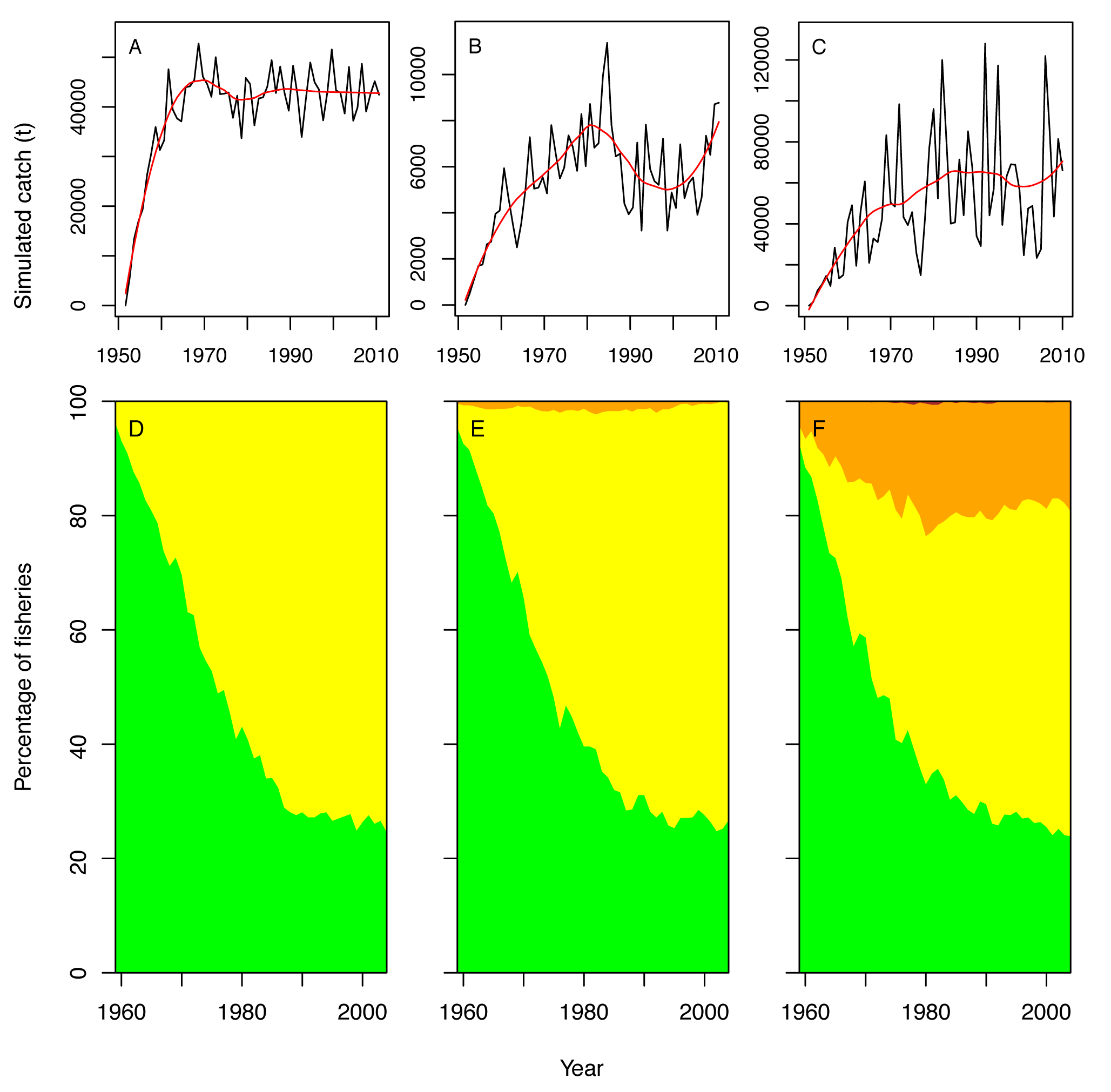

Supplement: Figure S7 — Demonstration of our fishery status assessment algorithm to simulated data. (A–C) Example of simulated increasing and then stationary catch series with multiplicative log-normal error about a random mean: log standard deviation of error of 0.10 (A), 0.25 (B), and 0.50 (C). Black lines indicate unfiltered catch. Red lines indicate loess smoothed fits. (D–F) Predicted stock status (expanding = green, fully exploited = yellow, over-exploited or restrictively managed = orange) from simulated data showing the robustness of our method to variability in the data as indicated for A, B, and C, respectively. (0.25 MB TIF) [file pone.0014735.s008.tif]

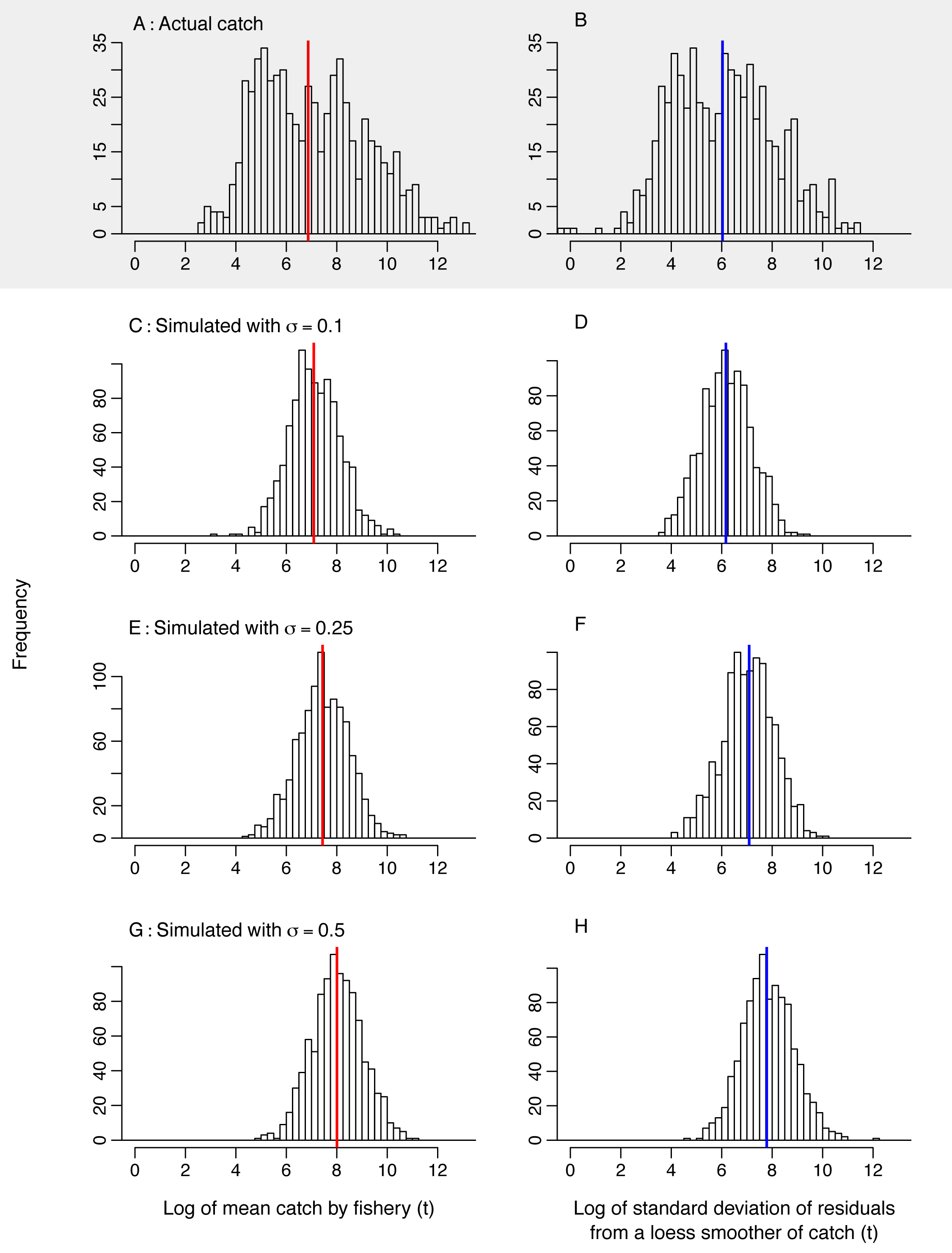

Supplement: Figure S8 — Characteristics of actual and simulated catch series. Frequency of log of mean catch values by fishery and log of the standard deviation of the residuals after fitting a loess smoother to each series (span = 0.5) from global invertebrate fisheries (A, B; grey background shading), and simulated series with σ = 0.1 (C, D), σ = 0.25 (E, F), and σ = 0.5 (G, H). See section Verification of fishery status estimation using simulated data for a description of σ. Red and blue vertical lines indicate median values. (0.39 MB TIF) [file pone.0014735.s009.tif]

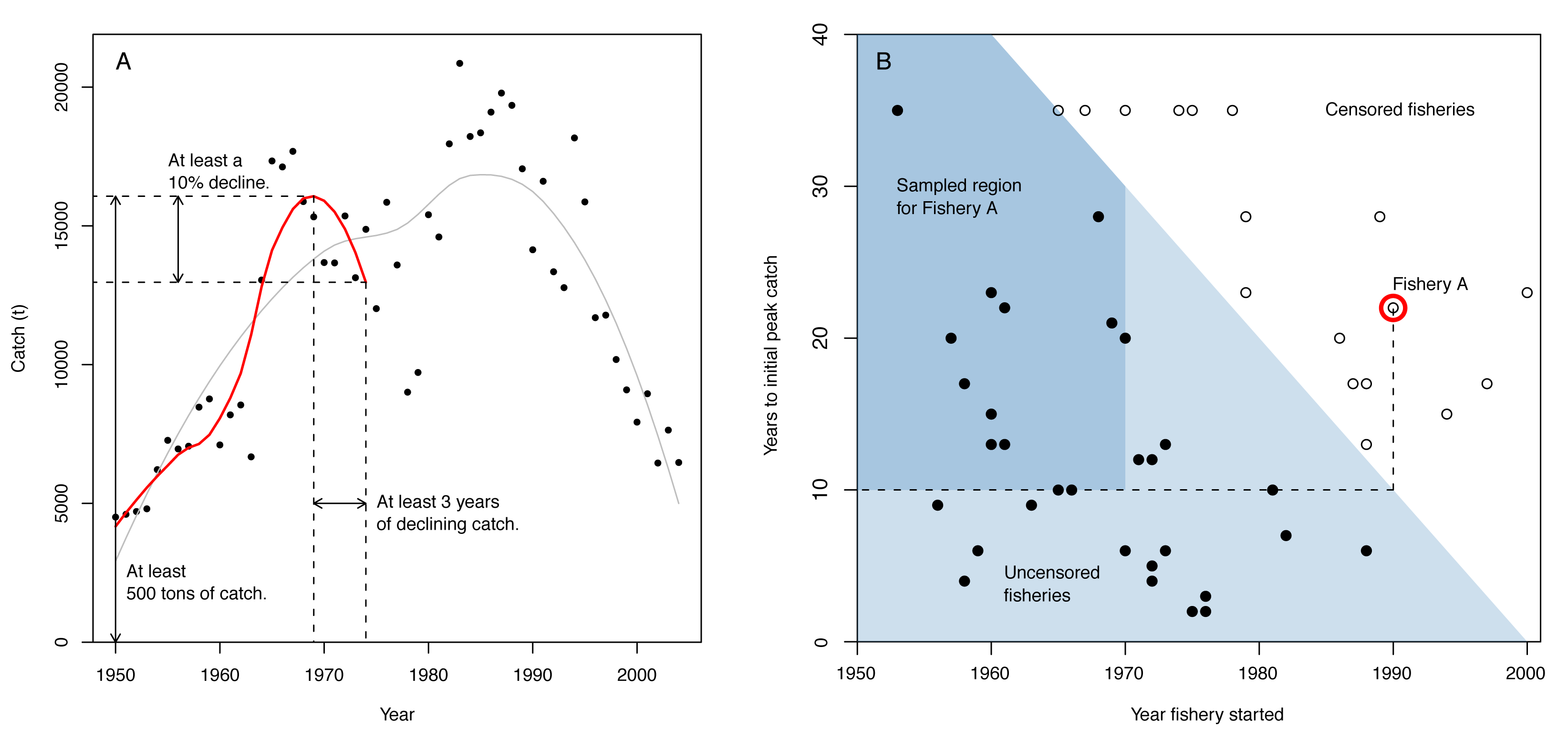

Supplement: Figure S9 — Methods used to determine years of initial peaks in catch. (A) Illustration of our algorithm for assigning year of initial peak catch. Dots represent raw catch values, grey line represents loess function fit through the entire catch series, and red line indicates loess function fit through data up to the year of initial peak catch. A fishery was considered to have peaked if there was at least 500 tonnes of catch, at least a 10% decline from peak catch, and at least 3 years of data after the peak in catch. This algorithm was applied dynamically each year until the first instance of peak catch was observed. (B) Illustration of sampling time to peak for one censored fishery (Fishery A, red circle). Fisheries for which time to peak could be calculated are shown with solid dots in the shaded blue triangle. Censored fisheries for which time to peak was sampled are shown with open dots. Vertical dashed line indicates known year in which Fishery A surpassed 10% of its maximum observed catch. Fishery A could therefore have been assigned a time to peak from any value above 10 years, as indicated by a horizontal dashed line, and before 1970 (dark blue shaded region). This sampling was repeated 1000 times. (0.25 MB TIF) [file pone.0014735.s010.tif]

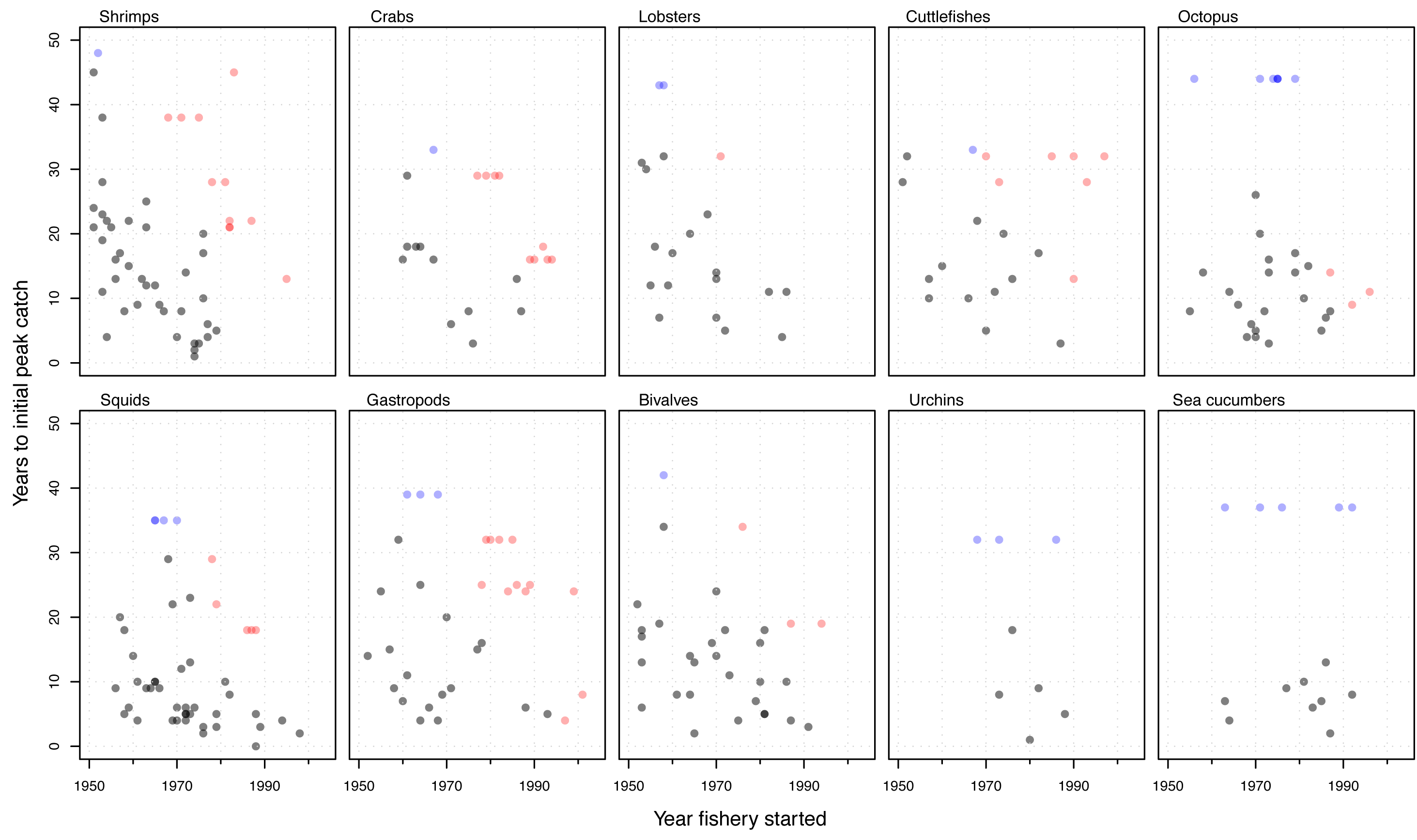

Supplement: Figure S10 — An example of time to peak catch vs. year of fishery initiation by taxonomic grouping for one random sampling of censored fisheries (red dots). Black dots represent known data points. In our analysis, the red dots were resampled 1000 times from possible time to peak values. Blue dots represent fisheries for which there were no fisheries to sample from. These were set to the maximum observed number of years for the earliest fishery affected (the left-most blue dot). (0.34 MB TIF) [file pone.0014735.s011.tif]
