# Supplementary material for: Rapid Global Expansion of Invertebrate Fisheries: Trends, Drivers, and Ecosystem Effects
Source: PLoS One. 2011 Mar 8;6(3):e14735. doi: 10.1371/journal.pone.0014735 (PMC3050978; doi:10.1371/journal.pone.0014735)
Supplement: Table S1 — Invertebrate catch for the 6 LMEs with the greatest total catch from 2000–2004. Also shown are the 3 taxonomic groups within each LME with the greatest catch. Catch values shown are annual averages over the 5-year span. LMEs are ordered by decreasing catch and within the LMEs the taxonomic groups are ordered by decreasing catch of that taxon. (0.03 MB PDF) [file pone.0014735.s012.pdf]

Table S1. Invertebrate catch for the 6 LMEs with the greatest total catch from 2000–2004. Also shown are the 3 taxonomic groups within each LME with the greatest catch. Catch values shown are annual averages over the 5-year span. LMEs are ordered by decreasing catch and within the LMEs the taxonomic groups are ordered by decreasing catch of that taxon.

LME name	Catch (t/sq. km)	Taxonomic group	Taxon catch (t/sq. km)
Yellow Sea	2.11	Shrimps and prawns	0.60
		Bivalves	0.53
		Crabs	0.34
East China Sea	1.67	Bivalves	0.50
		Shrimps and prawns	0.44
		Squids	0.24
N.E. U.S. Continental Shelf	1.9	Bivalves	1.55
		Crabs	0.13
		Lobsters	0.13
Newfoundland-Labrador Shelf	0.57	Shrimps and prawns	0.21
		Bivalves	0.20
		Crabs	0.12
South China Sea	0.52	Shrimps and prawns	0.17
		Bivalves	0.11
		Squids	0.07
Patagonian Shelf	0.47	Squids	0.39
		Bivalves	0.04
		Shrimps and prawns	0.04
