# Supplementary material for: Rapid Global Expansion of Invertebrate Fisheries: Trends, Drivers, and Ecosystem Effects
Source: PLoS One. 2011 Mar 8;6(3):e14735. doi: 10.1371/journal.pone.0014735 (PMC3050978; doi:10.1371/journal.pone.0014735)
Supplement: Table S2 — Distance and starting year of sea cucumber fisheries by country. Listed are each country's largest city (by population), its location, its distance from Hong Kong, the starting year of the sea cucumber fishery, and a verification reference. (0.09 MB PDF) [file pone.0014735.s013.pdf]

Table S2. Distance and starting year of sea cucumber fisheries by country. Listed are each country's largest city (by population), its location, its distance from Hong Kong, the starting year of the sea cucumber fishery, and a verification reference.

Country	Largest city	Latitude (°)	Longitude (°)	Distance (1000 km)	Start (year)	Reference
China/Hong Kong	HK Int. Airport	22.34	114.01	0	NA	NA
Philippines	Manila	14.62	120.97	0.87	1961	[1,2]
Indonesia	Jakarta	-6.18	106.83	1.34	1982	[3]
Malaysia	Kuala Lumpur	3.16	101.71	1.50	1963	[4]
Korea South	Seoul	37.56	126.99	1.67	1950	[5]
Japan	Tokyo	35.67	139.77	2.99	1950	[6]
Sri Lanka	Colombo	6.93	79.85	3.77	1976	[7]
Papua New Guinea	Port Moresby	-9.48	147.18	4.25	1986	[8]
Maldives	Male	4.17	73.50	4.45	1984	[9]
Solomon Islands	Honiara	-9.43	159.91	5.60	1984	[10]
New Caledonia	Noumea	-22.27	166.44	6.71	1977	[11]
Madagascar	Antananarivo	-18.89	47.51	6.92	1964	[12]
Fiji	Suva	-18.13	178.43	7.84	1970	[13,14]
Tanzania	Dar es Salaam	-6.82	39.28	8.05	1963	[15–17]
Egypt	Cairo	30.06	31.25	9.19	2002	[18,19]
US - Alaska	Anchorage	61.18	-149.19	11.28	1981	[20,21]
Canada West	Vancouver	49.28	-123.13	13.86	1985	[21,22]
US - Washington State	Seattle	47.62	-122.35	13.93	1971	[20,21]
US - California	Los Angeles	34.11	-118.41	14.24	1978	[20,21]
Mexico	Mexico City	19.43	-99.14	16.35	1998	[23,24]
Chile	Santiago	-33.46	-70.64	17.77	1992	[23]
Canada East	Halifax	44.67	-63.61	18.95	1999	[21,25,26]
US - Maine	Portland	43.66	-70.28	19.04	1993	[20,21]

References

1. Gamboa R, Gomez AL, Nievales MF (2004) The status of sea cucumber fishery and mariculture in the Philippines. In: Advances in sea cucumber aquaculture and management. FAO Fisheries Technical Paper 463, Rome, Italy: Food and Agriculture Organization of the United Nations. pp. 69–78.

2. Schoppe S (2000) Sea cucumber fishery in the Philippines. SPC Beche-de-mer Information Bulletin 13: 10–12.

3. Tuwo A (2004) Status of sea cucumber fisheries and farming in Indonesia. In: Advances in sea cucumber aquaculture and management. FAO Fisheries Technical Paper 463, Rome, Italy: Food

and Agriculture Organization of the United Nations. pp. 49–55.

4. Baine M, Sze CP (1999) Sea cucumber fisheries in Malaysia, towards a conservation strategy. SPC Beche-de-mer Information Bulletin 12: 6–10.

5. Choo P (2008) Population status, fisheries and trade of sea cucumbers in Asia. In: Sea cucumbers: A global review of fisheries and trade. Fisheries and Aquaculture Technical Paper 516, Rome, Italy: Food and Agriculture Organization of the United Nations. pp. 81–118.

6. Akamine J (2004) The status of the sea cucumber fisheries and trade in Japan: past and present. In: Advances in sea cucumber aquaculture and management, Rome, Italy: Food and Agriculture Organization of the United Nations. pp. 39–47.

7. Kumara PBTP, Cumaranathunga PRT, Linden O (2005) Present status of the sea cucumber fishery in southern Sri Lanka: A resource depleted industry. SPC Beche-de-mer Information Bulletin 22: 24–29.

8. Kinch J, Purcell S, Uthicke S, Friedman K (2008) Papua New Guinea: a hotspot of sea cucumber fisheries in the Western Central Pacific. In: Sea cucumbers: A global review of fisheries and trade. Fisheries and Aquaculture Technical Paper 516, Rome, Italy: Food and Agriculture Organization of the United Nations. pp. 57–57.

9. Joseph L (2005) Review of the beche de mer (sea cucumber) fishery in the Maldives. Technical Report 79, Food and Agriculture Organization of the United Nations, Madras, India.

10. Nash W, Ramofafia C (2006) Recent developments with the sea cucumber fishery in Solomon Islands. SPC Beche-de-mer Information Bulletin 23: 3–4.

11. Conand C, Byrne M (1993) A review of recent developments in the world sea cucumber fisheries. US Natl Mar Fish Serv Mar Fish Rev 55: 1–13.

12. Rasolofonirina R, Mara E, and MJ (2004) Sea cucumber fishery and mariculture in Madagascar, a case study of Toliara, southwest Madagascar. In: Advances in sea cucumber aquaculture and management. FAO Fisheries Technical Paper 463, Rome, Italy: Food and Agriculture Organization of the United Nations. pp. 133–149.

13. Uthicke S, Conand C (2005) Local examples of beche-de-mer overfishing: an initial summary and request for information. Beche-de-mer Information Bulletin 21: 9–14.

14. Ferdouse F (2004) World markets and trade flows of sea cucumber/beche-de-mer. In: Advances in sea cucumber aquaculture and management, Rome, Italy: Food and Agriculture Organization of the United Nations. pp. 101–116.

15. Mmbaga TK, Mgaya YD (2004) Sea cucumber fishery in Tanzania: identifying the gaps in resource inventory and management. In: Advances in sea cucumber aquaculture and management. FAO Fisheries Technical Paper 463, Rome, Italy: Food and Agriculture Organization of the United Nations. pp. 193–203.

16. Semesi A, Mgaya Y, Muruke M, Francis J, Mtolera M, et al. (1998) Coastal resources utilization and conservation issues in Bagamoyo, Tanzania. Ambio 27: 635–644.

17. Jiddawi N, Ohman M (2002) Marine fisheries in Tanzania. Ambio 31: 518–527.

18. Lawrence A, Ahmed M, Hanafy M, Gabr H, Ibrahim A, et al. (2004) Status of the sea cucumber fishery in the Red Sea – the Egyptian experience. In: Advances in sea cucumber aquaculture and management. FAO Fisheries Technical Paper 463, Rome, Italy: Food and Agriculture Organization of the United Nations. pp. 79–90.

19. Ahmed MI, Lawrence AJ (2007) The status of commercial sea cucumbers from Egypt's northern Red Sea Coast. SPC Beche de Mer Information Bulletin 26: 14–18.

20. Bruckner AW (2005) The recent status of sea cucumber fisheries in the continental United States of America. SPC Beche-de-mer Information Bulletin 22: 39–46.

21. Hamel JFH, Mercier A (2008) Population status, fisheries and trade of sea cucumbers in temperate areas of the Northern Hemisphere. In: Sea cucumbers: A global review of fisheries and trade. Fisheries and Aquaculture Technical Paper 516, Rome, Italy: Food and Agriculture Organization of the United Nations. pp. 257–291.

22. Hand CM, Hajas W, Duprey N, Lochead J, Deault J, et al. (2008) An evaluation of fishery and research data collected during the Phase 1 sea cucumber fishery in British Columbia, 1998 to 2007.

Canadian Science Advisory Secretariat Research Document 065, Fisheries and Oceans Canada, Nanaimo, BC, Canada.

23. Toral-Granda V (2008) Population status, fisheries and trade of sea cucumbers in Latin America and the Caribbean. In: Sea cucumbers: A global review of fisheries and trade. Fisheries and Aquaculture Technical Paper 516, Rome, Italy: Food and Agriculture Organization of the United Nations. pp. 213–229.

24. Ibarra AA, Soberón GR (2002) Economic reasons, ecological actions and social consequences in the Mexican sea cucumber fishery. SPC Beche-de-mer Information Bulletin 17: 33–36.

25. Rowe S, Comeau P, Singh R, Coffen-Smout S, Lundy M, et al. (2009) Assessment of the exploratory fishery for sea cucumber (*Cucumaria frondosa*) in southwest New Brunswick. Canadian Science Advisory Secretariat Research Document 005, Fisheries and Oceans Canada, Dartmouth, NS, Canada.

26. Therkildsen N, Petersen C (2006) A review of the emerging fishery for the sea cucumber *Cucumaria frondosa*: Biology, policy, and future prospects. SPC Beche-de-mer Information Bulletin 23: 16–25.
