# Supplementary material for: Rapid Global Expansion of Invertebrate Fisheries: Trends, Drivers, and Ecosystem Effects
Source: PLoS One. 2011 Mar 8;6(3):e14735. doi: 10.1371/journal.pone.0014735 (PMC3050978; doi:10.1371/journal.pone.0014735)
Supplement: Table S3 — Major gear groupings of gear categories from the Sea Around Us Project catch database. (0.03 MB PDF) [file pone.0014735.s014.pdf]

Table S3. Major gear groupings of gear categories from the Sea Around Us Project catch database.

Major gear grouping	Minor gear categories
Hand dredges and rakes	Hand dredges
	Raking devices
Lines and hooks	Lines
	Squid hooks
Diving and grasping	By diving
	Grasping with hand
	Tongs
	Without gear
Traps and pots	Box-like traps
	Traps
	Pots
Nets and midwater trawls	Driftnets
	Gillnets
	Ring nets
	Bagnets
	Purse seines
	Mid-water trawls
Benthic trawling and dredging	Bottom trawls
	Dredges
