# Supplementary material for: Rapid Global Expansion of Invertebrate Fisheries: Trends, Drivers, and Ecosystem Effects
Source: PLoS One. 2011 Mar 8;6(3):e14735. doi: 10.1371/journal.pone.0014735 (PMC3050978; doi:10.1371/journal.pone.0014735)
Supplement: Table S4 — Classification of invertebrate taxonomic groups into primary and secondary functional groups. Taxa are ordered approximately by decreasing trophic level. (0.05 MB PDF) [file pone.0014735.s015.pdf]

Table S4. Classification of invertebrate taxonomic groups into primary and secondary functional groups. Taxa are ordered approximately by decreasing trophic level.

Taxa	Primary	Secondary	Reference
Octopus	Prey, Predators		[1, 2]
Cuttlefishes	Prey, Predators		[1, 2]
Squids	Prey, Predators		[1, 2]
Sea stars	Prey, Predators	Scavengers	[2]
Shrimps and prawn	Prey, Predators	Herbivores, Filter feeders, Scavengers/Detritivores	[2, 3]
Crabs	Prey, Scavengers, Herbivores, Predators		[4]
Lobsters	Prey, Predators, Scavengers		[4]
Krill	Prey, Filter feeders		[3]
Urchins	Prey, Herbivores	Predators	[3]
Gastropods	Prey, Herbivores	Scavengers, Predators	[2]
Sea cucumbers	Prey, Detritivores, Filter feeders		[2, 3]
Bivalves	Prey, Filter feeders, Habitat	Detritivores	[2]

References

1. Boyle PR, Rodhouse P (2005) Cephalopods: Ecology and Fisheries. Wiley-Blackwell.

2. Hickman CP, Roberts LS, Larson A, l'Anson H, Eisenhour DJ (2006) Integrated Principles of Zoology. New York, NY, USA: McGraw-Hill, 13th edition.

3. Ruppert EE, Fox RS, Barnes RD (2004) Invertebrate zoology: a functional evolutionary approach. Belmont, CA, USA: Thomson-Brooks/Cole, 7th edition.

4. Pearse V, Pearse J, Buchsbaum M, Buchsbaum R (1987) Living invertebrates. Palo Alto, CA, USA: Blackwell Scientific Publications.
